# Targeting Glioblastoma via Selective Alteration of Mitochondrial Redox State

**DOI:** 10.3390/cancers14030485

**Published:** 2022-01-19

**Authors:** Akira Sumiyoshi, Sayaka Shibata, Zhivko Zhelev, Thomas Miller, Dessislava Lazarova, Ichio Aoki, Takayuki Obata, Tatsuya Higashi, Rumiana Bakalova

**Affiliations:** 1 Department of Molecular Imaging and Theranostics, National Institutes for Quantum and Radiological Science and Technology (QST), 4-9-1 Anagawa, Chiba 263-8555, Inage-ku, Japan; sumiyoshi.akira@qst.go.jp (A.S.); shibata.sayaka@qst.go.jp (S.S.); aoki.ichio@qst.go.jp (I.A.); obata.takayuki@qst.go.jp (T.O.); higashi.tatsuya@qst.go.jp (T.H.); 2 Faculty of Medicine, Trakia University, 6000 Stara Zagora, Bulgaria; zh_zhelev@yahoo.com; 3 Institute of Biophysics and Biomedical Engineering, Bulgarian Academy of Sciences, 1000 Sofia, Bulgaria; 4 IC-MedTech Corp., San Diego, CA 92101, USA; tmiller@ic-medtech.com; 5 Faculty of Medicine, Sofia University “St. Kliment Ohridski”, 1407 Sofia, Bulgaria; dessislaval@yahoo.com

**Keywords:** glioblastoma, redox targeting, mitochondrial redox cycling, tumor-associated ENOX2, TGF-β1, menadione, ascorbate

## Abstract

**Simple Summary:**

Glioblastoma is characterized by a pronounced redox imbalance due to elevated glycolytic and mitochondrial oxidative metabolism. New therapeutic strategies have been developed to modulate glioblastoma redox signaling to effectively suppress growth and prolong survival. However, drug selectivity and therapeutic relapse prove to be the major challenges. We describe a pharmacological strategy for the selective targeting and treatment of glioblastoma using the redox active combination drug menadione/ascorbate, which is characterized by tolerance to normal cells and tissues. Menadione/ascorbate treatment of glioblastoma mice suppressed tumor growth and significantly increased survival without adverse side effects. This is accompanied by increased oxidative stress, decreased reducing capacity and decreased cellular density in the tumor alone, as well as increased brain perfusion and decreased regulation of several oncoproteins and oncometabolites, which implies modulation of the immune response and reduced drug resistance. We believe that this therapeutic strategy is feasible and promising and deserves the attention of clinicians.

**Abstract:**

Glioblastoma is one of the most aggressive brain tumors, characterized by a pronounced redox imbalance, expressed in a high oxidative capacity of cancer cells due to their elevated glycolytic and mitochondrial oxidative metabolism. The assessment and modulation of the redox state of glioblastoma are crucial factors that can provide highly specific targeting and treatment. Our study describes a pharmacological strategy for targeting glioblastoma using a redox-active combination drug. The experiments were conducted in vivo on glioblastoma mice (intracranial model) and in vitro on cell lines (cancer and normal) treated with the redox cycling pair menadione/ascorbate (M/A). The following parameters were analyzed in vivo using MRI or ex vivo on tissue and blood specimens: tumor growth, survival, cerebral perfusion, cellular density, tissue redox state, expression of tumor-associated NADH oxidase (tNOX) and transforming growth factor-beta 1 (TGF-β1). Dose-dependent effects of M/A on cell viability, mitochondrial functionality, and redox homeostasis were evaluated in vitro. M/A treatment suppressed tumor growth and significantly increased survival without adverse side effects. This was accompanied by increased oxidative stress, decreased reducing capacity, and decreased cellular density in the tumor only, as well as increased cerebral perfusion and down-regulation of tNOX and TGF-β1. M/A induced selective cytotoxicity and overproduction of mitochondrial superoxide in isolated glioblastoma cells, but not in normal microglial cells. This was accompanied by a significant decrease in the over-reduced state of cancer cells and impairment of their “pro-oncogenic” functionality, assessed by dose-dependent decreases in: NADH, NAD^+^, succinate, glutathione, cellular reducing capacity, mitochondrial potential, steady-state ATP, and tNOX expression. The safety of M/A on normal cells was compromised by treatment with cerivastatin, a non-specific prenyltransferase inhibitor. In conclusion, M/A differentiates glioblastoma cells and tissues from normal cells and tissues by redox targeting, causing severe oxidative stress only in the tumor. The mechanism is complex and most likely involves prenylation of menadione in normal cells, but not in cancer cells, modulation of the immune response, a decrease in drug resistance, and a potential role in sensitizing glioblastoma to conventional chemotherapy.

## 1. Introduction

Glioblastoma is one of the most aggressive brain tumors. The rate of occurrence is 5 per 100,000 individuals, leading to 225,000 deaths per year globally [1]. The median survival is approximately 5 months after diagnosis for untreated patients and very rarely exceeds 18–22 months after therapy [1,2,3]. Only 5% of patients survive more than five years [4], and survival rates and mortality statistics for glioblastoma have remained unchanged for decades.

Surgical resection combined with radiotherapy and chemotherapy is the standard clinical protocol for this brain tumor, and temozolomide is still the “gold standard” for first-line chemotherapy of glioblastoma [5,6]. Temozolomide is a DNA-alkylating agent, which induces cell cycle arrest and apoptosis in cancer cells. It causes impressive suppression of tumor growth, but treatment with temozolomide is characterized by severe side effects [7,8]. In addition, DNA-alkylating anticancer drugs negatively affect all dividing normal cells in the organism such as immune, epithelial, and normal stem cells [9,10,11]. Some of these anticancer drugs also cause hemolysis [12]. This poses a risk of developing immune deficiency, anemia, and serious comorbidities because of chemotherapy.

Another major challenge in treating glioblastoma is how to avoid therapeutic relapse. Many advanced therapeutic strategies based on chemical or immunological mechanisms have been applied [1,13,14], but the clinical effects are still modest in terms of median survival and recurrence. Enormous efforts and costs have been invested to discover new molecular targets for stopping the therapeutic resistance, as well as progression and invasion, of brain tumors [15]. However, currently available therapies have a palliative effect. The development and progression of glioblastoma, as well as its resistance to standard therapy, remain unclear.

Disruption of redox signaling and induction of redox imbalance in the cells and tissues, accompanied by moderate chronic oxidative stress, are decisive factors in triggering carcinogenesis, including in the brain [16,17,18,19]. Brain tissues are vulnerable to oxidative damage due to their high demand for oxygen and energy provided by high mitochondrial activity [20]. It is widely accepted that oxidative stress causes mitochondrial dysfunction and “vice versa”, dysfunctional mitochondria are one of the main endogenous sources of oxidative stress in cancer because of incomplete coupling of electrons and H^+^ with oxygen in the electron transport chain (ETC), contributing to further production of reactive oxygen species (ROS) [21,22]. Mitochondrial signaling directs various vital intracellular processes such as aerobic respiration, apoptosis, cell proliferation and survival, nucleic acid synthesis, and oxidative stress itself—all associated with cancer progression. The role of mitoepigenetic regulation in cancer cells and the potential employment of dysfunctional mitochondria as valuable anticancer targets were recently described by Chen et al. in their comprehensive review article [23]. In addition, tolerated oxidative stress in cancer is a consequence of increased basal metabolic activity and peroxisome activity, uncontrolled growth factors of cytokine signaling, oncogene activity, and enhanced activity of oxidase and oxygenase enzymes such as NADPH oxidase complex (NOX), cyclooxygenases, and lipoxygenases [24,25]. Cancer cells also express on their surface a unique tumor-associated hydroquinone (NADH) oxidase with protein disulfide–thiol exchange activity (tNOX/ENOX2), which contributes to the generation of ROS (mainly superoxide) and induction of oxidative stress [26,27]. This protein marker is a subject of interest in our study.

Glioblastoma is characterized by pronounced redox imbalance [28,29], expressed in the high oxidative capacity of cancer cells due to their elevated glycolytic and mitochondrial oxidative metabolism [30,31]. Glioblastoma cells rely on glycolysis as a source of energy but easily adapt to bioenergetic stress by activating and using their mitochondria and oxidative phosphorylation (OXPHOS) to survive and grow [32]. Thus, these cells break out of metabolic restrictions and adapt to their microenvironment. Recently, it was reported that mitochondrial fatty acid oxidation (mFAO) is overexpressed in these cells [33,34]. They use fatty acids as a fuel for energy production and perhaps for other reasons, especially when glucose is not sufficient. Abnormal mitochondrial functionality has been linked to the development of glioblastoma [32,33,34]. These findings imply that the assessment and modulation of the redox state and function of cancerous mitochondria are key factors that may provide highly specific targeting and treatment of this brain cancer.

ROS/RNS (superoxide, hydrogen peroxide, other hydroperoxides, nitric oxide, etc.) and reducing equivalents (NADH, NADPH, antioxidants, etc.) are often described as “redox-active compounds”, and the balance between them as the “redox status”, “redox state”, or “bioreduction capacity” of cells, tissues, and body fluids [35]. Changes in their spatial and temporal distribution play a central role in carcinogenesis [36]. Currently, the redox state is considered an important diagnostic marker and a therapeutic target for all pathologies associated with a disturbance in cellular redox signaling. The tissue redox state is analyzed by using exogenous redox-sensitive molecular probes and imaging techniques. This methodological approach shows that early-stage glioblastoma in animals is characterized by a high reducing capacity, and the same is true for the tissues of healthy individuals [29,37]. In contrast, the microenvironment of moderate- and advanced-stage glioblastoma is characterized by decreased reducing capacity and increased oxidative activity compared to healthy tissues [29,37], which contributes to genomic instability [24,38].

The oxidative activity of cancer cells and tissues is due to abnormal ROS/RNS levels and not necessarily associated with high oxygen tension. On the contrary, hypoxia is a hallmark of solid tumors such as glioblastoma. Hypoxic signaling pathways have important implications for the adaptation of cancer cells to oxidative stress, the induction of uncontrolled proliferation, and immortalization. The correlation between oxidative stress, hypoxia, and resistance of glioblastoma tumors to radiotherapy is sufficiently described in Torrisi et al. [39].

It has also been shown that cancer progression affects the redox state of healthy tissues distant from the primary tumor locus [29,37]. The oxidative capacity of these tissues increases, and they become vulnerable to oxidative stress and damage [29]. This could be crucial to inducing side effects, tumor invasion, and resistance to therapy [29,37].

It is well known that redox imbalance and oxidative stress contribute to inflammation [21,24,25]. Some of the most frequently discussed endogenous substances, responsible for the spread of inflammation away from the primary tumor and affecting the redox homeostasis of healthy tissues, are proinflammatory cytokines produced by tumor-associated macrophages and fibroblasts [40,41]. Our study considers the role of transforming growth factor beta complex 1 (TGF-β1) in glioblastoma, as a prominent proinflammatory cytokine known to contribute to the spread of inflammation, fibrosis, and metastasis [42].

Many studies now clearly demonstrate that the redox environment plays an important role in the initiation, progression, and regression of glioblastoma, and new targeted redox therapies should be the focus of scientists, pharmacists, and clinicians. In the last decade, new therapeutic strategies have been designed to modulate redox signaling in this brain tumor, to improve immune recognition and immune response, and to potentiate the effect of standard therapy [28,43,44,45]. Most of the redox-active compounds found to be cytotoxic to glioblastoma cells are characterized by overproduction of ROS, including overproduction of mitochondrial ROS, a decrease in mitochondrial potential, and/or depletion of glutathione (Table S1, see the Supporting Materials). However, most of them have not been studied for selectivity towards glioblastoma cells only, and off-target effects limit their prospects.

In this article, we describe highly selective targeting and treatment of glioblastoma in vitro and in vivo, using the redox-active combination drug menadione/ascorbate (M/A, 1/100 mol/mol ratio). It has been empirically established that at a ratio of 1/100 (mol/mol), this combination exhibits the strongest synergistic cytotoxicity towards cancer cells compared to other ratios such as 1/10 and 1/50. This effect is thought to be due to the redox cycling between the two molecules in the cells and the extracellular environment, which requires an excess of ascorbate and leads to the overproduction of ROS (Figure S1, see the Supporting Materials). The mechanism and the combination index are described in many articles summarized recently in [46]. M/A has attracted the attention of researchers due to the synergistic anticancer effect of the two molecules, as well as its ability to kill cancer cells without affecting the viability of normal cells [46]. Importantly, the strong synergistic antiproliferative and cytotoxic effect on cancer cells is inherent for the combination of ascorbate and menadione (pro-vitamin K3), but not for the combination of ascorbate and vitamin K1 or K2 [46]. This suggests that the prenylation of menadione and its conversion into vitamin K1 or K2 will decrease its anticancer activity in combination with ascorbate. In this context, inhibitors of prenyltransferase and the mevalonate pathway should potentiate the effect of M/A. However, it is not known how this triple combination will affect the homeostasis of normal cells and tissues, and whether this mechanism underlies the selective cytotoxicity of M/A against cancer cells only.

Menadione and ascorbate are known to interfere with the mitochondrial ETC. Studies have demonstrated that menadione and other quinones affect mitochondrial respiration directly and even provide explanations for the molecular mechanisms of this mitochondrial interference [47,48,49]. For example, it has been demonstrated that pharmacological ascorbate and menadione are beneficial in the treatment of mitochondrial diseases, bypassing Complex I and Complex III deficiency [47,49,50]. The combination of ascorbate and menadione has been included in the “List of Dietary Supplements for Primary Mitochondrial Disorders” by the U.S. Department of Health and Human Services, National Institute of Health (NIH). Menadione and ascorbate have been applied as a dietary supplement in combination with coenzyme Q10, niacin, riboflavin, and thiamin to bypass Complex I and Complex III of the mitochondria [51].

Experiments on animal models demonstrated that the anticancer effect of M/A is apparent at low, pharmacologically achievable doses. This differs significantly from the vitamin activity of the two molecules. Studies also suggest that ascorbate should not be considered simply as a pro-oxidant or antioxidant [52,53]. Ascorbate is one of the most abundant cytosolic redox-active compounds and could serve as a “buffer” of excess-reducing equivalents in the intracellular aqueous phase of cancer cells due to their oxidative environment. Steady-state levels of ascorbate are maintained by the NADH-dependent cytochrome b5 reductase 3 (Cyb5R3) [54,55], and they are significantly higher in cancer cells compared to normal cells due to overexpression of vitamin C transporters (glucose transporter 1 (GLUT1) and sodium-dependent vitamin C transporters 1 and 2 (SVCT1, SVCT2)) [56].

Menadione is known to cross the blood–brain barrier (BBB) [57]. Ascorbate has been shown to cross the BBB via GLUT1 in its oxidized form and via SVCT1 and SVCT2 in its oxidized and reduced forms [57,58,59]. Brain tissues contain some of the highest ascorbic acid concentrations among mammalian tissues. Intracellular ascorbate is involved in the physiology of the nervous system, including the processes of differentiation, maturation, and neuronal survival, modulation of neurotransmission, myeline formation, protection against glutamate toxicity, and others [58,59].

The aim of the present study was to elucidate the possibility of targeting and treating glioblastoma with the redox-active combination drug menadione/ascorbate by selectively altering the redox state of cancerous (dysfunctional) mitochondria. The experiments were conducted in vitro on human glioblastoma cells (U87MG) and normal microglial cells, as well as in vivo on glioblastoma-bearing mice (U87MG intracranial model). Our efforts were directed to clarifying: (i) the molecular mechanism(s) for recognizing dysfunctional mitochondria by M/A and altering their redox state in glioblastoma cells and tissues, without significantly affecting the homeostasis of healthy cells and tissues; (ii) the role of two key proteins, tNOX and TGF-β1, for the induction of oxidative stress in the tumor, as well as for changing the redox state of healthy tissues and their vulnerability to oxidative damage. Up-regulation of tNOX and/or TGF-β1 has been found to correlate with a poor prognosis and low survival in patients with glioblastoma [26,27,42,60]. The effect of M/A treatment on cerebral blood flow (CBF) and the apparent diffusion coefficient (ADC) was also analyzed by using MRI in vivo. In recent years, these parameters have been accepted as potential non-invasive biomarkers for monitoring and predicting the response of brain tumors to anticancer therapy and their malignancy by using multiparametric MRI [61,62].

## 2. Materials and Methods

### 2.1. Chemicals

L-Ascorbic acid and menadione were purchased from Sigma-Aldrich (Weinheim, Germany).

All reagents used in the experiments were “analytical grade” or “HPLC grade”.

### 2.2. Cells and Treatment Protocol

The experiments were performed on normal and cancer cell lines, purchased from ATCC^®^, Washington, DC, USA (U87MG, EOC2).

U87MG cells were cultured in DMEM (Sigma-Aldrich, Weinheim, Germany), supplemented with 10% FBS and antibiotics (100 U/mL penicillin and 100 μg/mL streptomycin). EOC2 cells were cultured in DMEM, supplemented with 10% FBS. Cells were cultured in a humidified atmosphere at 37 °C, saturated with 5% CO_2_.

To remove the adhesive cells from the plates, we used a trypsin/EDTA solution (0.05% of trypsin/EDTA; Sigma-Aldrich, Weinheim, Germany) for U87MG, and a cell scraper for EOC2.

The cells were collected by centrifugation (125× *g* for 10 min) and placed in a fresh medium without antibiotics prior to treatment with the respective substance. The cells (3 × 10^5^ cells/mL) were incubated with the drug for different time intervals in a cell incubator. At each time interval, aliquots were used for analyses.

Ascorbate was dissolved in PBS (10 mM, pH 7.4). Menadione was dissolved in dimethyl sulfoxide (DMSO; Sigma-Aldrich, Weinheim, Germany) to 10 mM stock solutions, and then several working solutions in PBS were prepared. The final concentration of DMSO in the cell suspension was below 1%. At this concentration, DMSO did not affect cell viability.

### 2.3. Cell Proliferation and Viability Assays

Cell proliferation and viability were analyzed using CellTiter-Glo^TM^ Luminescent Cell Viability Assay (Promega, Madison, WI, USA).

Briefly, 100 μL aliquots of cell suspensions were placed in 96-well plates and incubated with M/A for 24 and 48 h, in a humidified atmosphere (at 37 °C, 5% CO_2_). An amount of 100 μL of CellTiter-Glo reagent (containing luciferin and luciferase) was added to each well, followed by incubation using the protocol recommended by the manufacturer. The luminescence, produced by the luciferase-catalyzed conversion of luciferin into oxyluciferin by living cells, was detected using a microplate reader (TECAN Infinite^®^ M1000, Vienna, Austria), working in a chemiluminescent mode.

The linear range for this assay was up to 5 × 10^5^ cells per well.

### 2.4. Apoptosis Assay

The induction of apoptosis was analyzed by the expression of phosphatidylserine (PSer) on the cell surface, using FITC-Annexin V Apoptosis Detection Kit (BioVision, Milpitas, CA, USA). Briefly, the cells (5 × 10^5^ cells/mL) were incubated with M/A for 48 h in a humidified atmosphere. The cell medium was removed, and 100 μL of PBS containing 2.5 mM of CaCl_2_ (annexin V-binding buffer) and 5 μL of fluorescein isothiocyanate (FITC)-annexin V were added to each well and incubated for 10 min at room temperature in the dark. The cells were washed three times with annexin V-binding buffer and resuspended in the same buffer. FITC-annexin V bound to PSer exposed on the cell surface was detected spectrofluorimetrically at λ_ex_ = 488 nm and λ_em_ = 535 nm, using a microplate reader (TECAN Infinite^®^ M1000, Vienna, Austria).

### 2.5. Mitochondrial Superoxide Assay

MitoSOX™ Red Mitochondrial Superoxide Indicator (Molecular Probes, Invitrogen, Oregon, OR, USA) is a fluorogenic probe for highly selective detection of superoxide in the mitochondria of live cells. The probe is a dihydroethidium derivate, containing a triphenylphosphonium group. Once in the mitochondria, MitoSOX™ Red reagent is oxidized by superoxide and exhibits red fluorescence. The probe is not oxidized by other ROS/RNS, and its oxidation is prevented by superoxide dismutase [63].

Briefly, MitoSOX™ Red was dissolved in DMSO to a 5 mM stock solution, which was diluted with Hank’s Balanced Salt Solution (HBSS, containing Ca^2+^ and Mg^2+^) to prepare 3 μM MitoSOX™ Red working solution on the day of the experiment. One milliliter of cells (5 × 10^5^ cells/mL) was collected by centrifugation, and the pellet was resuspended in 1 mL of 3 μM MitoSOX™ Red. The samples were incubated for 30 min at room temperature, protected from light, washed three times with PBS using centrifugation, and finally resuspended in 1 mL of PBS. The fluorescence intensity was detected immediately at λ_ex_ = 510 nm and λ_em_ = 580 nm, using a microplate reader (TECAN Infinite^®^ M1000, Vienna, Austria).

### 2.6. Mitochondrial Membrane Potential

The mitochondrial membrane potential was analyzed using tetramethylrhodamine methyl ester (TMRE; Sigma-Aldrich, Weinheim, Germany) as described in Levraut et al. [64], with slight modifications. TMRE is a cell-penetrating, cationic fluorophore, which accumulates in the mitochondrial matrix based on the mitochondrial membrane potential. The fluorescence intensity is proportional to the mitochondrial potential and decreases upon depolarization of the mitochondrial membrane.

Briefly, 1 mL of cells (5 × 10^5^ cells/mL) were placed in 12-well plates. An amount of 5 μL of TMRE (from a 40 μM stock solution in DMSO) was added to each well. The samples were incubated at 37 °C for 30 min, washed twice with PBS using centrifugation, and finally resuspended in 1 mL of PBS. The fluorescence intensity was detected immediately at λ_ex_ = 550 nm and λ_em_ = 575 nm, using a microplate reader (TECAN Infinite^®^ M1000, Vienna, Austria).

### 2.7. Succinate Assay

The succinate level was analyzed using Succinate Assay Kit (Colorimetric) (Abcam, Tokyo, Japan). The analysis is based on a coupled enzyme reaction, which results in a colored product with absorbance reaching a maximum at 450 nm, which is proportional to the succinate concentration in the sample. Succinate was used as a standard.

Briefly, cells (1 × 10^6^ cells per sample) were lysed in 100 μL of succinate assay buffer as described in the manufacturer’s instructions. An amount of 50 μL (in duplicates) of each cell lysate was placed in a 96-well plate and incubated with 50 μL of reaction mix-1 or 50 μL of reaction mix-2 (without succinate converter; blank sample) for 20 min at 37 °C, in the dark. Absorbance at 450 nm was recorded, using a microplate reader (TECAN Infinite^®^ M1000, Vienna, Austria). A blank sample was included to correct the NADH-dependent background absorbance.

### 2.8. NAD^+^/NADH Quantification Assay

The NAD^+^/NADH level was analyzed using NAD^+^/NADH Quantification kit (Sigma-Aldrich, St. Louis, MO, USA). This assay is specific for NAD^+^ and NADH and does not detect NADP^+^ and NADPH. NAD_total_ and NADH are quantified spectrophotometrically at 450 nm.

Briefly, cells (2 × 10^5^ cells per sample) were placed in 100 μL NAD^+^/NADH extraction buffer and homogenized as described in the manufacturer’s instructions. Cell lysates were purified on a 10 kDa cut-off spin filter. An amount of 50 μL (in duplicates) of each sample was placed in a 96-well plate and incubated with 100 μL of master reaction mix for 5 min at room temperature to convert NAD^+^ to NADH (for NAD_total_ determination). An amount of 10 μL of NADH developer was added to each sample and incubated for 1 h at room temperature. Absorbance at 450 nm was recorded, using a microplate reader (TECAN Infinite^®^ M1000, Vienna, Austria). For detection of NADH only, aliquots of cell lysates were placed in a heating block for 30 min at 60 °C, to decompose NAD^+^, before proceeding to analysis.

### 2.9. Total Glutathione Assay

The total glutathione (GSH/GSSG) in cell suspensions (5 × 10^6^ cells/mL) was analyzed by OxiSelect™ Total Glutathione (GSSG/GSH) Assay kit (Cell Biolabs, Inc., San Diego, CA, USA) as described in the manufacturer’s instructions. The method is based on the reduction of GSSG to GSH by glutathione reductase in the presence of NADPH and the subsequent addition of chromogen. Chromogen reacts with the thiol group of GSH, with the production of a spectrophotometrically detectable compound at 405 nm, using a microplate reader (TECAN Infinite^®^ M1000, Vienna, Austria). The total glutathione content in the cell suspension was determined by a calibration curve using a glutathione standard.

### 2.10. Total Antioxidant Capacity (TAC) Assay

The TAC assay was performed on cell and tissue lysates using OxiSelect^TM^ Total Antioxidant Capacity (TAC) Assay kit (Cell Biolabs, Inc., San Diego, CA, USA). The method is based on the reduction of Cu^2+^ to Cu^+^ by antioxidants and other reducing equivalents in the biological sample. Cu^+^ interacts with a chromophore to obtain a color product with an absorption maximum at 490 nm. The value of absorption is proportional to the total antioxidant and reducing capacity of the biological object.

Briefly, cell and tissue lysates were prepared as described in the manufacturer’s instructions. All lysates were adjusted to the same protein concentration, and 20 μL of aliquots was placed in a 96-well plate. Each sample was incubated with a copper ion reagent and chromophore as described in the instruction. The absorption of the product at 490 nm was detected by a microplate reader (TECAN Infinite^®^ M1000, Vienna, Austria). Three independent experiments were performed for each lysate, with two parallel sample measurements for each experiment.

The total antioxidant capacity of the samples was determined by a calibration curve using uric acid as a standard. The results are presented as “Total Antioxidant Capacity (TAC)”, which is equivalent to “Total Reducing Capacity” in “mM Uric Acid Equivalents”. An amount of 1 mM of uric acid corresponds to 2189 μM of Cu^2+^-reducing equivalents.

### 2.11. tNOX (ENOX2) Assay

Ecto-NOX disulfide-thiol exchanger 2 (ENOX2, tNOX) expression was detected in cell and tissue lysates using Human ENOX2 ELISA kit (Cusabio, Huston, TX, USA) and Mouse ENOX2 ELISA kit (LifeSpan BioScience, Seattle, WA, USA), respectively. Cell and tissue lysates were prepared and analyzed as described in the manufacturer’s instructions. The protein concentration in the lysates was determined by Bradford analysis. Both assays are based on the quantitative sandwich enzyme immunoassay technique. The antigen–antibody complex was detected spectrophotometrically at 450 nm, based on the oxidation of 3,3′5,5′-tetramethylbenzidine (TMB) by horseradish peroxidase conjugated to avidin, which interacts with the biotinylated secondary antibody in the sandwich. Lyophilized tNOX (ENOX2) protein was used as a standard. All samples were run in triplicate.

### 2.12. TGF-β1 Assay

Transforming growth factor-beta 1 (TGF-β1) expression was detected in plasma using Human TGF-β1 ELISA kit (Funakoshi, Tokyo, Japan) and Mouse TGF-β1 ELISA kit (Abcam, Tokyo, Japan). Samples were collected and stored at −80 °C for approximately one month before the analysis of TGF-β1 and then analyzed, as described in the manufacturer’s instructions.

The assay is based on the quantitative sandwich enzyme immunoassay technique. The antigen–antibody complex was detected spectrophotometrically at 450 nm (and 570 nm as a reference wavelength), based on the oxidation of 3,3′5,5′-tetramethylbenzidine (TMB) by horseradish peroxidase conjugated to streptavidin, which interacts with the biotinylated secondary antibody in the sandwich. Lyophilized TGF-β1 protein was used as a standard. All samples were run in triplicate.

### 2.13. Animals and Treatment Protocol

The animal experiments in this study were approved by the National Institutes for Quantum Science and Technology (QST) Institutional Animal Care and Use Committee, Chiba, Japan, and all experiments were performed in accordance with relevant guidelines and regulations.

BALB/c nude mice were obtained from Charles River Labs (Tokyo, Japan). All mice were male, used at 6–8 weeks of age, and maintained in specific pathogen-free conditions.

U87MG glioblastoma model: The experimental design is shown in Figure S2 (in the Supporting Materials). Human glioblastoma U87MG cells (1 × 10^5^ in 2 μL per mouse) were inoculated into the brain of anesthetized mice using a stereotaxic device (SR-6M, Narishige, Tokyo Japan). The cell suspension was injected 2 mm to the right of and 1 mm anterior from the bregma, at a depth of 3 mm within 2 min. The glioma-bearing mice were assigned to the following two groups: (i) control group—single intracranial injection of saline solution; (ii) M/A-treated group—single intracranial injection of 70 μg/7 mg of M/A per kg body weight. The volume of all intracranial injections of M/A was 5 μL. The mice in the M/A-treated group were also subjected to oral administration of M/A in the drinking water (150 mg/15 g of M/A per 1 L; freshly prepared every day except on weekends). The mice in the control group received M/A-free water. One day before cell transplantation, the mice were placed on a vitamin C- and menadione-deficient diet (CLEA, Tokyo, Japan).

Before injection of the drug, the tumor was visualized in the brain of each mouse using T_2_-weighted (T_2_W) MRI, and the initial tumor size was calculated. Body weight was measured once or twice per week. MRI measurements were also performed once or twice per week depending on parameters analyzed. The approved humane endpoint was two months after cell transplantation. However, the mice were sacrificed at the following conditions: when the tumor size exceeded 100 mm^3^, or at rapid weight loss of 25%, headedness, and/or tetraplegia.

### 2.14. Measurement of Hemoglobin, Hematocrit, and Thrombosis

Hb and Hct were measured by a Start Strip Xpress2 device (Nova Biomedical, Waltham, MA, USA), and prothrombin time was measured by test trips for the CoaguChek^®^ XS system (Roche Diagnostics K.K., Tokyo, Japan) using peripheral blood taken from the tail (after disinfection) of mice anesthetized with isoflurane. The presence of thrombosis was assessed by light microscopy.

### 2.15. In Vivo MRI Measurements

Each mouse was anesthetized with isoflurane (3% for initial induction and 1–2% during MRI scanning) and was placed in the prone position on a custom-built MRI stage with a bite bar and a facemask. The respiration rate was monitored using a respiration sensor (SA Instruments, Inc., New York, NY, USA) and was regulated at 80–120 breaths per minute. The core body temperature was monitored with a rectal probe (FOT-M and FTI-10, FISO Technologies Inc., Quebec, Canada) and was regulated at 37.0 ± 1.0 °C using a water-circulating pad and a warm circulation air system. MRI data were acquired using a horizontal 7.0 T Bruker BioSpec 70/40 MRI system with an 86 mm volume transmit and a 4-channel phased array receive-only cryoprobe (Bruker Biospin, Ettlingen, Germany). The software and console of the MRI scanner were ParaVision 360 and AVANCE NEO, respectively. Following the standard adjustment routines, pilot scans (Tripilot sequence) were used for accurate positioning of the animal head inside the magnet.

The T_2_W images were obtained using a spin echo 2D-RARE (rapid acquisition with relaxation enhancement) pulse sequence with the following parameters: repetition time = 3000 ms, effective echo time = 60 ms, RARE factor = 8, field of view = 16 × 16 mm^2^, matrix size = 160 × 160, in-plane resolution = 0.1 × 0.1 mm^2^, number of slices = 13, slice thickness = 0.3 mm, slice gap = 0 mm, fat suppression = on, and number of averages = 8.

CBF images were obtained using a 2D-FAIR (flow-sensitive alternating inversion recovery) RARE pulse sequence with the following parameters: inversion recovery time = 100, 300, 500, 700, 900, 1100, 1300, 1500, 1700, and 1900 ms, repetition time = 12,000 ms, effective echo time = 4.54 ms, RARE factor = 20, field of view = 16 × 16 mm^2^, matrix size = 80 × 80, in-plane resolution = 0.2 × 0.2 mm^2^, number of slices = 1, slice thickness = 0.75 mm, and number of averages = 1.

ADC images were obtained using a diffusion (2D-DtiStandard Diffusion Tensor Imaging Standard) pulse sequence with the following parameters: diffusion gradient duration = 2.5 ms, diffusion gradient separation = 8.4 ms, diffusion b-value = 800 s/mm^2^, repetition time = 2500 ms, echo time = 17.5 ms, field of view = 16 × 16 mm^2^, matrix size = 160 × 160, in-plane resolution = 0.1 × 0.1 mm^2^, number of slices = 1, slice thickness = 0.75 mm, and number of averages = 1.

MRI data analysis was performed using custom-written software in MATLAB (MathWorks, Natick, MA, USA). CBF image analysis consists of calculations of the selective T_1_ map (T1_sel_), calculation of the global T_1_ map (T1_nonsel_), and calculation of the perfusion map (CBF). The calculations of T1_sel_ and T1_nonsel_ were performed using a nonlinear least square fit to the data for each voxel in the images with different inversion recovery times (10 images in each). CBF was calculated from the measurements of T1_sel_ and T1_nonsel_, which were obtained using the equation: CBF (mL/100 g/min) = λ * T1_nonsel_/T1_blood_ * (1000/T1_sel_ − 1000/T1_nonsel_), where λ is the blood–brain partition coefficient, i.e., the ratio between the water concentration per gram of brain tissue and per milliliter of blood. λ was set to 4980. T1_blood_ was set to 2.3, which was derived from the measurements of rat blood at 7.0 T. ADC images were obtained using the equation: ADC (mm^2^/s) = 1/b_1_ * ln[SI(b_0_)/SI(b_1_)], where SI(b_0_) and SI(b_1_) are the image intensities obtained by two gradient b-values, b_0_ = 0 and b_1_ = 800 s/mm^2^. For the region-of-interest (ROI) analysis, the image segmentation was performed with ITK-SNAP software and custom-written software in MATLAB. 

For redox imaging, five control images of the mouse brain were taken before injection with the following parameters: T_1_-weighted (T_1_W) incoherent gradient echo sequence (FLASH), repetition time = 75 ms; echo time = 3.5 ms; flip angle = 45 degrees; field of view = 3.2 × 3.2 cm; number of averages = 4; scan time = 19.6 s; matrix = 64 × 64; slice thickness = 1.0 mm; and number of slices = 4. A solution of a nitroxide derivative (multi-spin mito-TEMPO dissolved in PBS, pH 7.4) was injected via the tail vein (100 μL per 25 g mouse; 0.4 μmol/g b.w.) 100 sec after beginning the scan. T_1_W images were acquired continuously within approximately 14 min, using the parameters described above. The final dose of nitroxide was much lower than the LD_50_ value calculated for i.v. administration in nude mice. The averaged value of the first five control sequences (recorded before injection of nitroxide) was calculated, and each sequence of the kinetic measurement was normalized to this averaged value.

### 2.16. Statistical Analysis

All results are expressed as the mean ± standard deviation (SD). The normality of the distribution for all parameters of each experimental group in vivo was initially confirmed by using the Kolmogorov–Smirnov test. The most extreme differences for all experimental groups were below the critical D-values. Based on the normality of the distribution in all groups, the comparisons between them were performed using Student’s *t*-test for multiple comparisons. Two-tailed *p*-values of less than 0.05 were considered statistically significant.

## 3. Results

### 3.1. Effect of M/A on Tumor Growth and Survival of Glioblastoma-Bearing Mice

The mice were inoculated with U87MG cells, and after 7 days, tumors were visualized in the animals, using MRI. The initial tumor size was approximately 15–20 mm^3^. The mice were divided into two groups: M/A-treated group—single intracranial injection of M/A plus daily oral administration of M/A in the drinking water; control group—single intracranial injection of saline solution.

**Figure 1:**
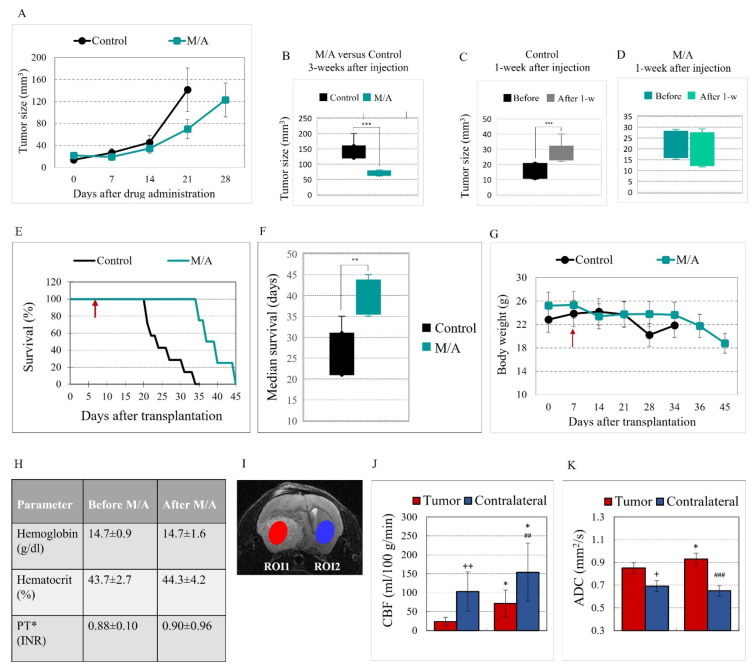
(**A**) Effect of single intracranial injection (5 μL) of menadione/ascorbate (M/A) on tumor growth in the brain of U87MG glioblastoma-grafted mice, detected by T_2_-weighted magnetic resonance imaging (MRI) within 35 days after cell transplantation and 28 days after drug administration (70 μg/7 mg of M/A per kg body weight). The drug was administered on day 7 of the brain cell transplant, when the tumor size was 14.17 ± 4.5 mm^3^ in the control group and 22.0 ± 6.4 mm^3^ in the M/A-treated group. Number of mice in each experimental group: control group (*n* = 7); M/A-treated group (*n* = 5). Data are the mean ± SD from 3 mice at each time point. (**B**) Comparison of tumor size between control group and M/A-treated group, measured 3 weeks after intracranial injection of saline solution or M/A injection (*** *p* < 0.001 versus control group). (**C**) Comparison of tumor size in the control group, detected before and 1 week after intracranial injection of saline solution (*** *p* < 0.001 versus before injection). (**D**) Comparison of tumor size in the M/A-treated group, detected before and 1 week after intracranial injection of M/A. (**E**) Effect of single intracranial injection of M/A on survival of U87MG glioblastoma-grafted mice. Red arrow indicates the time of injection (day 7 after cell transplantation). Data are the mean ± SD from 7 mice in the control group and 5 mice in the M/A-treated group. (**F**) Median survival of mice in the groups described in (**E**) (** *p* < 0.01 versus control group). Data are the mean ± SD from 7 mice in the control group and 5 mice in the M/A-treated group. (**G**) Dynamics of body weight of control and M/A-treated glioblastoma-grafted mice. Red arrow indicates the time of injection (day 7 after cell transplantation). At each time point, the data are the mean ± SD from 2–7 mice in the control group and 2–5 mice in the M/A-treated group, depending on their survival. (**H**) Hematological parameters analyzed in healthy mice before and after intravenous administration of M/A (140 μg/14 mg per kg body weight). * INR—international normalized ratio (reference value ≤ 1.1); PT—prothrombin time. Data are the mean ± SD from 3 mice in each group with three measurements for each specimen in the case of Hb and Hct, and six measurements for each specimen in the case of INR. (**I**–**K**) Multiparametric MRI analysis in M/A-treated and untreated glioblastoma mice (U87MG intracranial model). (**I**) Definition of regions of interest (tumor area and contralateral hemisphere). (**J**) Effect of M/A on cerebral perfusion (cerebral blood flow, CBF) and (**K**) apparent diffusion coefficient (ADC). Absolute values of CBF and ADC were detected in the tumor area and contralateral hemisphere of the brain. Both parameters were analyzed 21 days after cell transplantation and 14 days after drug administration. Data are the mean ± SD from 3 mice in each group with two measurements (slices) per mouse. * *p* < 0.05, M/A-treated tumor or contralateral hemisphere versus subsequent untreated (control) tumor or contralateral hemisphere; ^++^ *p* < 0.01 for CBF and ^+^ *p* < 0.05 for ADC, contralateral hemisphere versus tumor hemisphere in untreated mice; ^##^ *p* < 0.01 for CBF and ^###^ *p* < 0.001 for ADC, contralateral hemisphere versus tumor hemisphere in M/A-treated mice.

The tumor size was measured each week after cell transplantation (Figure 1A). In the M/A-treated group, tumor growth was significantly slower compared to the control group (Figure 1A,B). In the control group, there was a 2-fold increase in the tumor size for a week (*p* < 0.001; Figure 1C), while single intracranial injection of M/A stopped tumor growth for a week (Figure 1D). M/A-treated mice had longer survival than the control mice (Figure 1E). Median survival was 39.3 ± 4.3 days for the M/A-treated group versus 25.9 ± 5.4 days for the control group (*p* < 0.01; Figure 1F). The body weight in both experimental groups was relatively stable (Figure 1G). M/A-treated mice lost weight after 34 days of cell transplantation.

Single intracranial injection and oral administration of M/A in glioblastoma-bearing mice did not affect the levels of hemoglobin (Hb) and hematocrit (Hct) (Figure 1H). Hemolysis and erythropenia were not detected even after single intravenous injection of M/A (in doses up to 140 μg/14 mg per kg body weight) in mice, which exceeds twice the intracranial dose used in our study. Intravenous injection of M/A at the same dose did not affect the prothrombin time (Figure 1H) and did not lead to thrombosis (Figure S3 in the Supporting Materials). All hematological parameters were within the respective reference values before and after M/A treatment. This is direct evidence that menadione does not act as a coagulant when used in combination with ascorbate in doses with a pronounced anticancer effect. M/A at concentrations up to 20/2000 μM/μM, exceeding those that can be achieved pharmacologically, did not affect the viability of normal human lymphocytes and normal human epithelial cells, which was analyzed in vitro in our previous study [46,65].

### 3.2. Effect of M/A on Brain Perfusion and Cell Density of Glioblastoma-Bearing Mice

Using multiparametric MRI analysis, we assessed the effects of M/A on CBF and ADC, characterizing the homeostasis of the brain tumors and their potential susceptibility to therapy. Two regions of interest (ROIs) were defined in the brain for this analysis: (i) tumor area; (ii) contralateral hemisphere (Figure 1I). The values of CBF and ADC in both ROIs of the untreated (control) and M/A-treated glioblastoma mice are shown in Figure 1J,K. A significant increase in CBF values was observed in both ROIs of the M/A-treated group compared to the untreated group (~3 times in the tumor area and ~50% in the contralateral hemisphere) (Figure 1K). M/A treatment increased the values of ADC in the tumor area (~15%, *p* < 0.01), but not in the contralateral hemisphere, compared to the untreated glioblastoma mice (Figure 1I).

### 3.3. Effect of M/A on Tissue Redox State of Glioblastoma-Bearing Mice

**Figure 2:**
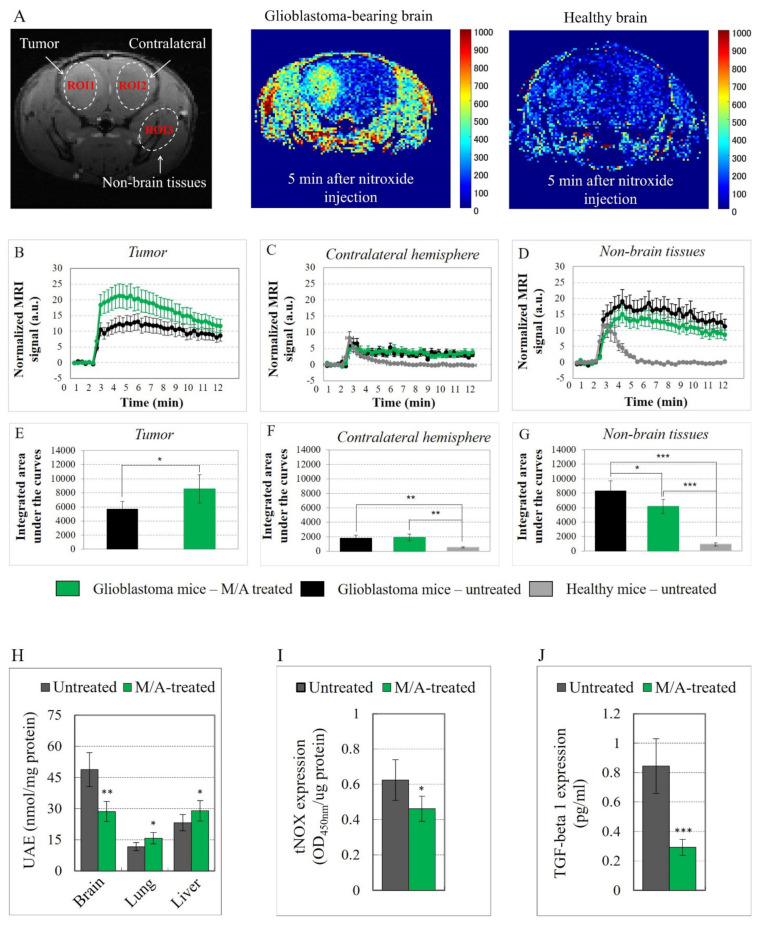
Redox imaging in glioblastoma-bearing mice using nitroxide-enhanced MRI. (**A**) Definition of regions of interest (ROIs): ROI1—tumor area; ROI2—contralateral hemisphere; ROI3—non-brain tissues (black and white image), and representative nitroxide-enhanced magnetic resonance images of glioblastoma-bearing brain and healthy brain, obtained 5 min after intravenous injection of nitroxide probe (mito-TEMPO)—a marker of tissue redox activity (color images). Color images—calculated extracted MRI signal obtained after injection of nitroxide probe and normalized to the baseline (native) signal obtained before injection. (**B**–**D**) Kinetic curves of nitroxide-enhanced MRI signal in U87MG glioblastoma-grafted mice (M/A treated and untreated) and healthy untreated mice. Kinetic curves were analyzed 7 and 14 days after drug administration. Data are the mean ± SD from 6 mice in each group. (**G**–**I**) Integrated areas under the kinetic curves shown in D, E, and F, respectively. * *p* < 0.05, ** *p* < 0.01, *** *p* < 0.001. (**H**) Total reducing capacity of tissues isolated from untreated and M/A-treated glioblastoma-bearing mice. (**I**,**J**) Expression of tNOX (**I**) and TGF-β1 (**J**) in the brain tissue and serum, respectively, isolated from untreated and M/A-treated glioblastoma-bearing mice. In (**B**–**G**), data are the mean ± SD from 3 mice in each group with three measurements (slices) per mouse. In (**H**–**J**), data are the mean ± SD from 3 mice in each group with three measurements for each specimen. Tissue and blood specimens were collected 1 week after the start of M/A administration and 2 weeks after cell transplantation. Treatment of mice was the same as described in Figure 1. * *p* < 0.05, ** *p* < 0.01, *** *p* < 0.001 versus the untreated group.

The tissue redox state was analyzed and visualized by the dynamic nitroxide-enhanced MRI before and after intravenous administration of multi-spin mito-TEMPO as a redox-sensitive probe. Mito-TEMPO penetrates the blood–brain barrier and cell membrane and is localized mainly in the mitochondria [66]. Three ROIs were selected in the glioma-bearing mice: (i) the tumor area in the brain (ROI1); (ii) the contralateral non-cancerous hemisphere (ROI2); and (iii) the surrounding (non-brain) tissues (ROI3) (Figure 2A). ROI2 and ROI3 were selected for comparison in untreated healthy mice. In glioblastoma mice, the signal intensity was high and long lived in the tumor area and non-brain surrounding tissues (Figure 2A—color image). In healthy mice, the nitroxide-enhanced MRI signal disappeared within 5 min after the injection of the nitroxide probe (Figure 2A—color image).

The kinetic curves of the normalized MRI signals in the respective ROIs are shown in Figure 2B–D. In healthy mice, the signal increased after the injection of the nitroxide probe, followed by a rapid decrease to the baseline in both ROIs (Figure 2C,D—gray curves). The enhancement of the MRI signal in the beginning is due to the presence of a nitroxide radical in the bloodstream and its penetration and accumulation in the subsequent tissue, whereas the decrease is due to its reduction to non-contrast hydroxylamine, which occurs predominantly in cells. In this case, the half-life of the nitroxide-enhanced MRI signal (τ_1/2_) was approximately 80 s, and the duration of the signal was approximately 5–6 min (Figure 2D—gray curves). The profile of the histograms indicates a high reducing capacity of healthy tissues for the nitroxide probe.

In glioblastoma-bearing mice, the nitroxide-enhanced MRI signal in the tumor (Figure 2B) and other ROIs (Figure 2C,D) had completely different kinetics than in healthy mice. The signal increased after injection and then reached a plateau without a decrease to the baseline within 14 min. The signal intensity was significantly higher in the tumor area than in the contralateral hemisphere (Figure 2B,C). A high nitroxide-enhanced MRI signal was also found in the non-brain tissues that are located relatively far from the primary tumor locus (Figure 2D—black and green curves). The histograms indicate a high oxidative activity in the tumor and non-tumor tissues of the glioblastoma-bearing mice.

The integrated areas under the curves are shown in Figure 2E–G. Tissue oxidative activity in glioblastoma-bearing mice (M/A treated and untreated) was significantly higher than in healthy mice. M/A treatment led to a further increase in oxidative activity in the tumor tissue, but remarkably not in the contralateral hemisphere. Rather, M/A treatment decreased the oxidative activity in the non-brain tissues of glioblastoma-bearing mice.

We also analyzed tissue total redox capacity (TRC) ex vivo in the brain, as well as in other organs distant from the primary tumor locus—liver and lungs—isolated from M/A-treated and untreated glioblastoma-bearing mice (Figure 2H). The results correlate with those of the redox imaging in vivo. The TRC of the brain tissues of M/A-treated mice was ~40% lower compared to the untreated controls, while the liver and lung tissues of M/A-treated mice were characterized by a ~20% higher reducing capacity compared to the untreated glioblastoma mice. This is indirect evidence that M/A increases the level of oxidative stress in tumor tissue but decreases the level of oxidative stress in healthy tissues of glioblastoma mice.

M/A treatment of glioblastoma mice also resulted in a slight but significant decrease in tNOX expression in the brain tissues (Figure 2I), and a strong decrease (~3-fold) in TGF-β1 release in the blood (Figure 2J).

### 3.4. Effect of M/A on Mitochondrial Functionality, tNOX Expression, and Prenylation

**Figure 3:**
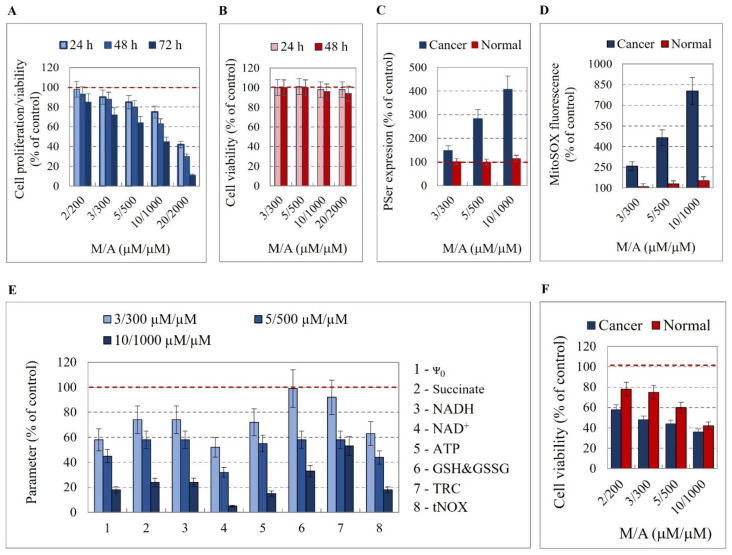
Effects of menadione/ascorbate (M/A) on mitochondrial and cellular redox state and viability. (**A**) Concentration-dependent and time-dependent effects of M/A on proliferation and viability of glioblastoma cells (U87MG). (**B**) Concentration-dependent and time-dependent effects of M/A on viability of normal microglial cells (EOC2). (**C**) Concentration-dependent effect of M/A on the induction of apoptosis in glioblastoma (blue columns) and normal microglial cells (red columns) after 48 h of incubation. (**D**) Concentration-dependent effects of M/A on steady-state levels of mitochondrial superoxide in normal (red columns) and glioblastoma cells (blue columns), analyzed after 48 h of incubation in humidified atmosphere. (**E**) Concentration-dependent effects of M/A on parameters representative of mitochondrial and cellular redox homeostasis: mitochondrial membrane potential (ψ_0_), succinate, NADH, NAD^+^, total glutathione (GSH and GSSG), total reducing capacity (TRC), and tumor-associated NADH oxidase (tNOX). All parameters were analyzed after incubation of glioblastoma cells (U87MG) with M/A for 48 h in humidified atmosphere. (**F**) Effect of cerivastatin (5 μM) on viability of M/A-treated glioblastoma cells (U87MG) and normal microglial cells (EOC2) after 48 h of incubation. The cells in the control sample were treated with cerivastatin only. The initial number of cells in all samples was 0.6 × 10^5^ cells per well. Data are the mean ± SD from three independent experiments with two parallel measurements for each experiment in (**A**–**D**,**F**), and two independent experiments with four parallel measurements for each experiment in (**E**). In all charts, the value of each parameter in the untreated (control) samples was considered as 100% (red dashed lines). All differences exceeding 15% were statistically significant.

We compared the effects of M/A treatment on the viability and redox homeostasis of isolated human glioblastoma cells (U87MG) and normal microglial cells (EOC2) (Figure 3). Cells were treated with different concentrations of M/A for 24, 48, and 72 h, and cell proliferation and viability were analyzed (Figure 3A,B). M/A was applied at low/tolerable concentrations (<5/500 μM/μM) and high concentrations (≥5/500 μM/μM). At concentrations <5/500 μM/μM, M/A suppresses cell proliferation without inducing cell death, while at concentrations ≥ 5/500 μM/μM, M/A induces cell death, which has been demonstrated in vitro on cancer cell lines other than glioblastoma [46,67,68,69].

In normal microglial cells, M/A did not affect cell viability up to 20/2000 μM/μM (Figure 3B). In glioblastoma cells, M/A exhibited a strong cytotoxicity at concentrations ≥ 5/500 μM/μM, with an IC_50_ value of ~8/800 μM/μM (Figure 3A). At low/tolerable concentrations (2/200 and 3/300 μM/μM), M/A markedly suppressed cell proliferation without significant cell death. The selective cytotoxicity of M/A on glioblastoma cells only was also confirmed by the expression of PSer on the cell surface as a marker for induction of apoptosis (Figure 3C—blue columns versus red columns). The combination induced dose-dependent overproduction of mitochondrial superoxide in glioblastoma cells—5 to 8 times above the level in untreated cells (Figure 3D, blue columns). In normal microglial cells, M/A induced a relatively low increase in mitochondrial superoxide and mild oxidative stress, which seems to be well tolerated (Figure 3D, red columns). The comparative analysis showed that M/A exhibits selective cytotoxicity towards glioblastoma cells without significantly affecting the viability of normal microglial cells. This targeted cytotoxic effect of M/A was accompanied by induction of severe oxidative stress in glioblastoma cells only.

M/A treatment of glioblastoma cells induced a significant dose-dependent decrease in the values of the parameters characterizing mitochondrial functionality and cellular redox homeostasis: mitochondrial membrane potential, NADH, NAD^+^, oncometabolite succinate, steady-state ATP amount, total intracellular glutathione, total cellular reducing capacity, and tNOX (Figure 3E). These parameters were not analyzed in M/A-treated normal microglial cells, as M/A did not affect the viability of these cells at the concentrations used and did not induce apoptosis, and the effect on mitochondrial superoxide was negligible (Figure 3B–D—red columns). An interesting finding was that cerivastatin—a specific inhibitor of mevalonate pathways and a non-specific prenyltransferase inhibitor [70]—potentiated the cytotoxic effect of M/A on glioblastoma cells but compromised the safety of M/A on normal microglial cells, inducing significant cytotoxicity in these cells when combined with M/A (Figure 3F). This suggests that the prenylation of menadione and/or key proteins in the cells is essential for the targeted anticancer effect of the M/A combination, which is discussed below.

## 4. Discussion

Summarizing, our study demonstrates that the redox pair “menadione/ascorbate” inhibited the growth and viability of glioblastoma cells and tissues without adversely affecting the viability of normal cells and tissues at pharmacologically achievable concentrations. These findings were obtained in vivo on glioblastoma mice (Figure 1 and Figure 2) and in vitro on cultured cells (Figure 3).

A single intracranial injection of M/A (70 μg/7 mg per kg body weight) in glioblastoma mice and subsequent oral administration in the drinking water (15 g/150 mg per liter) significantly increased survival without adverse drug-related side effects (Figure 1E).

Tissues (cancerous and healthy) of glioblastoma mice were characterized by a higher oxidative capacity than tissues of healthy mice (Figure 2A–I). M/A treatment significantly increased oxidative stress and decreased the reducing capacity of the tumor tissue (Figure 2B,E) but did not affect the redox state of healthy brain tissues (Figure 2C,F). Furthermore, M/A treatment decreased oxidative stress in non-brain tissues distant from the primary tumor (such as liver and lung tissue) and increased their reducing capacity (Figure 2D,G).

M/A treatment inhibited the expression of TGF-β1 (Figure 2J) and tNOX in the glioblastoma mice (Figure 2I).

M/A treatment decreased the cell density (evaluated by ADC) in the tumor tissue but not in non-tumor brain tissues (Figure 1K) and increased perfusion (evaluated by CBF) markedly in the tumor and slightly in the non-tumor brain tissues (Figure 1J).

M/A induced cytotoxicity and apoptosis in isolated glioblastoma cells but not in normal microglial cells (Figure 3A–C). This effect was accompanied by dose-dependent overproduction of mitochondrial superoxide in glioblastoma cells only (Figure 3D), as well as by a dose-dependent decrease in the levels of the following parameters: mitochondrial membrane potential, NADH, NAD^+^, succinate, steady-state ATP, intracellular glutathione, total cellular reducing capacity, and tNOX (Figure 3E). This indicates an impairment of the mitochondrial function and cellular redox homeostasis of glioblastoma cells.

The above observations suggest that M/A may differentiate cancer cells and tissues from healthy ones. Thus, the drug causes oxidative stress only in the tumor, evaluated by redox imaging using MRI in vivo and the TRC assay ex vivo. It does not adversely affect the redox state in healthy tissues (such as lung and liver tissue) and may even suppress oxidative stress in them (Figure 2H). The TRC assay, also called the “Total Antioxidant Capacity Assay”, used in our study provides partial information on the antioxidant status in cells and tissues. The study of the effects of M/A on the state of various endogenous pro-oxidants, antioxidants and other reducing equivalents would help to elucidate the fine molecular mechanism(s) of its redox-modulating effect.

We also found that M/A treatment does not cause hemolysis, erythropenia, and thrombosis at the selected doses (Figure 1H). The harmless and even beneficial effects of M/A on normal cells and healthy tissues may be one of the reasons for the good tolerance to the drug.

A few articles have described the effect of menadione and ascorbate on the viability of glioma/glioblastoma cells in vitro. Menadione alone and in combination with ascorbate has been shown to significantly inhibit DNA replication and the growth of patient-derived glioma cells [57]. The authors reported that M/A completely inhibited the re-growth of glioma cells at long-term treatment in vitro, while menadione-treated cells resumed their proliferation. Ascorbate administered alone in tolerable doses (≤2.5 mM) did not suppress the growth of human glioma cells [57]. However, high doses of ascorbate (≥5 mM) significantly increased intracellular ROS and decreased the proliferation of human glioma cells, causing necrotic cell death [71]. Intravenous administration of ascorbate (1 g and 2 g per kg body weight) to glioblastoma-bearing rats inhibited tumor growth and invasion, analyzed ex vivo [71]. It should be noted that the risk of adverse side effects is potentially high at these doses of ascorbate, according to the literature [72,73,74]. Recently, it has been reported that pharmacological ascorbate has a radio-protective effect on glioma-bearing mice (GL261 intracranial model) [75]. The authors found that tumor growth was faster, and survival was shorter, in mice treated with 6 Gy radiation plus ascorbate versus those treated with 6 Gy radiation alone. It is obvious that menadione and ascorbate, administered alone, do not have the power of the anticancer effect of their combination in glioma/glioblastoma cells and tissues. Studies on experimental animals have reported that oral and parenteral M/A potentiates the efficiency of conventional chemotherapy and radiotherapy of cancer in vivo and inhibits invasion and metastasis [76,77,78,79,80]. This suggests that the molecular mechanisms of action of the M/A combination are different from those of ascorbate administered alone.

Menadione and ascorbate are powerful redox cyclers and, when administered alone and especially in combination, induce intracellular production of ROS by interaction with molecular oxygen [46,81,82]. It is generally accepted that M/A causes cancer cell death by induction of oxidative stress and subsequent replicative stress [67,68,69]. This mechanism has also been described in M/A-treated glioma cells in vitro [57]. However, it does not explain the selective cytotoxicity of M/A towards cancer cells only or the possible role in the immune system

**Figure 4:**
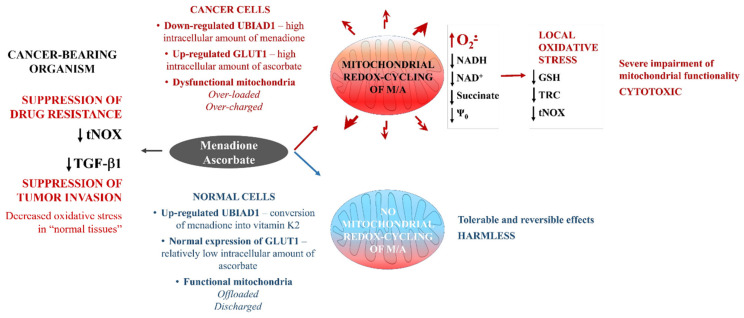
Potential molecular mechanism(s) for selective targeting of cancer cells by M/A via specific redox cycling in the overloaded and overcharged cancerous mitochondria due to down-regulation of UBIAD1 and inhibition of menadione prenylation, and up-regulation of vitamin C transporters in cancer cells. Role of tNOX and TGF-β1 in M/A-mediated anticancer effect on cancer-bearing organisms.

We propose another potential mechanism for the selective targeting of cancer cells by M/A (Figure 4). Cancer cells, including glioblastoma cells, differ significantly from normal cells in several characteristics. Cancer cells have overexpressed ascorbate transporters (GLUT1, SVCT1, and SVCT2) [56], which allows the accumulation of much higher concentrations of ascorbate in them compared to normal cells. Cancer cells are overloaded with reducing equivalents, such as NADH and succinate, and their mitochondria are overcharged due to the high CoQ10H_2_/CoQ10 ratio [24,52,53,83,84]. This allows a highly specific and accelerated redox cycling of M/A in dysfunctional cancerous mitochondria, but not in normally functioning mitochondria. Normal cells express UbiA prenyltransferase domain containing protein 1 (UBIAD1), also known as transitional epithelial response protein 1 (TERE1), which converts menadione to vitamin K2 [85,86]. Recently, we established that the combination vitamin K2/ascorbate (≤20/2000 μM/μM) has a negligible effect on cell viability [46]. However, down-regulation of UBIAD1 is a hallmark of multiple cancers, according to the Human Protein Atlas [87], and this prenyltransferase is one of the newest prognostic markers discussed in cancer, according to recent cohort studies [87,88,89]. The conversion of menadione to vitamin K2 should be suppressed in cancer cells due to down-regulation of UBIAD1. Thus, cancer cells should accumulate high concentrations of menadione/ascorbate, while normal cells should contain a vitamin K2/ascorbate combination. In our study, we found that the safety of M/A on normal cells was compromised by treatment with cerivastatin, a specific inhibitor of mevalonate pathways and a non-specific prenyltransferase inhibitor (Figure 3F). This is indirect evidence that UBIAD1 could be a key factor in the selective cytotoxicity of M/A to cancer cells, and this is the subject of ongoing study in our team.

UBIAD1/TERE1 is known as a tumor suppressor gene [85,86,87,88,89]. Down-regulation of UBIAD1 has been shown to activate Ras-MAPK signaling [90], one of the main signaling networks in gliomas [91]. UBIAD1 is also involved in the regulation of cholesterol levels via steroid and xenobiotic receptor (SXR) target genes, as well as the synthesis of ubiquinones [92,93]—decisive factors in inducing carcinogenesis and survival of cancer cells.

Another possible mechanism of action of M/A in glioblastoma could be related to the effect of ascorbate on histone methylation. Vitamin C has been found to play a pivotal role in remodeling the epigenome by enhancing the activity of Jumonji-C domain-containing histone demethylases (JHDMs) and the ten-eleven translocation (TET) proteins [94]. The ability of vitamin C to potentiate the activity of histones and DNA-demethylating enzymes has clinical application in the treatment of cancer. Vitamin C deficiency has been widely reported in cancer patients and has been shown to accelerate cancer progression in animal models [94]. However, it should be noted that the effect of vitamin C on demethylation of histones may antagonize the anticancer effect of DNA-alkylating drugs, such as temozolomide. MGMT promoter methylation is the key mechanism of MGMT gene silencing and predicts a favorable outcome in patients with glioblastoma who are exposed to alkylating agent chemotherapy [95,96]. We found that M/A did not abolish or decrease the cytotoxic effect of temozolomide in isolated glioblastoma cells (Figure S4, see the Supporting Materials). There was a very slight potentiation of the cytotoxicity of temozolomide at low/tolerable concentrations of M/A, but not at high concentrations. This indicates that the effect of ascorbate (when it is in combination with menadione) on histone demethylation is not critical for the anticancer effect of temozolomide.

Based on these factors, we assume that the targeted anticancer effect of M/A is due to its specific redox cycling in the dysfunctional mitochondria of cancer cells only, which is accompanied by severe oxidative stress and an impairment of their “pro-oncogenic” functionality. This mechanism appears to be valid for glioblastoma. M/A treatment of isolated glioblastoma cells decreased their over-reduced state (decrease in NADH, succinate, glutathione, etc.) and mitochondrial membrane potential, which was accompanied by overproduction of mitochondrial superoxide (Figure 3D,E). We assume that in normal cells, there is no such mitochondrial redox cycling between the two substances and the effects of M/A are tolerable and reversible (Figure 4). Our in vivo data support this hypothesis. M/A treatment of glioblastoma-bearing mice induced severe oxidative stress in the tumor tissue, but not in the non-tumor tissues (Figure 2A–G).

The mechanism of glioblastoma targeting by M/A is complex and likely not limited simply to modulating the redox state and overproduction of ROS in cancer cells and tissues. The decreased expression of tNOX and TGF-β1 (Figure 2I,J) and the decreased production of the oncometabolite succinate (Figure 3E) in M/A-treated animals suggest an influence of the immune response. The expression and activity of tNOX have been shown to have a direct effect on the immune response in cancer. Recently, Hsieh et al. administered an anti-tNOX vaccine to mice with lung cancer [97]. The authors reported that vaccinated mice showed significantly less tumor mass and a significantly lower histological outcome of the lesion. They concluded that tNOX has an immunogenic effect, and that the tNOX vaccine has potential in the immunotherapy of lung cancer. However, it should be noted that the effect of the tNOX vaccine on tumor growth is modest. The cytokine TGF-β also plays a crucial role in regulating the immune response and cancer progression, which is sufficiently described in two recent review articles [98,99]. TGF-β exerts systemic immune suppression and inhibits the recognition of cancer cells by the host immune system. Suppression of TGF-β expression or inhibition of its conversion from the latent to the active state leads to: (i) an increased anticancer immune response mediated by CD8+ T cells and natural killer cells; (ii) increased neutrophil-attracting chemokines, resulting in recruitment and activation of neutrophils with an anticancer phenotype. This cytokine regulates the infiltration of inflammatory/immune cells and cancer-associated fibroblasts in the tumor microenvironment, causing direct changes in cancer cells. TGF-β has been shown to directly affect PD-1 expression on T cells and PD-L1 expression on cancer cells, which suppresses the anticancer activity of the native immune system [100]. Another important immunomodulator is the oncometabolite succinate, which was sufficiently analyzed and described by Ryan et al. in the context of carcinogenesis [101].

tNOX is a hydroquinone (NADH) oxidase that serves as a terminal oxidase in a process whereby protons and electrons are shuttled from the inside of the cell across the cell membrane to molecular oxygen at the cell surface (plasma membrane electron transport) [102]. Being located on the cell surface and without membrane anchoring domains, the protein is released from the cells and appears in the serum and urine as a relatively stable and readily accessible biofluid marker [103]. tNOX is a molecule involved in the hallmarks of cancer cells, down-regulated in slow-proliferating non-cancer cells, and currently undiscovered in non-proliferating normal cells [26,27,102]. Thus, suppression of tNOX with anticancer drugs could selectively inhibit cell growth and induce apoptosis in cancer cells, but not in normal cells [103,104,105,106,107]. Some conventional anticancer drugs have been shown to transiently up-regulate tNOX expression, thereby enhancing the migration of cancer cells and causing the development of drug resistance [107,108]. Inhibition of tNOX expression/activity has been recently reported to affect mitochondrial function by decreasing NAD^+^-dependent SIRT1 deacetylase activity and increasing acetylation of p53, which causes augmentation of ROS-dependent mitochondrial autophagia and induces apoptosis in cancer cells [103,104,105,106]. The down-regulation of tNOX and the decreased NADH and NAD^+^ levels in M/A-treated glioblastoma cells and tissues observed in our study (Figure 2I and Figure 3E) are indirect evidence that this mechanism could be important in glioblastoma. In this context, tNOX could be a valuable therapeutic target, distinguishing cancer cells from normal cells and enabling selective damage of cancerous mitochondria. tNOX could also maintain the level of the quinone form of the menadione required for the intracellular redox cycling with ascorbate in cancerous mitochondria.

TGF-β is another molecular target in glioblastoma, whose expression was strongly suppressed in M/A-treated mice (Figure 2J). In general, TGF-β has a complex role in carcinogenesis including cancer cell motility and metastasis, proliferation of tumor-associated fibroblasts, epithelial-to-mesenchymal transition, angiogenesis, and immunosuppression [109]. TGF-β is responsible for the spread of inflammation, and its expression in glioblastoma-bearing mice may explain, at least partially, the high oxidative capacity of healthy tissues distant from the primary tumor locus (Figure 2D,G). The spread of inflammation is always associated with the spread of ROS generation and oxidative stress [24,110]. Other proinflammatory cytokines (TNF-alpha, IL-2, IL-12, etc.) are also of great interest as therapeutic targets for cancer [111] that could be affected by M/A. However, this hypothesis needs verification.

What could be the possible mechanism of the spread of inflammation far from the primary tumor and the effect of M/A treatment?

Transplantation of cancer cells in the brain can be considered an “inflammatory signal” [112]. The xenograft leads to a local migration and an activation of a wide variety of immune cells in the target tissue, especially in the microenvironment of the primary transplant. This activation may trigger redox imbalance due to the “oxidative burst” of the immune cells and the production and release of ROS in the grafted area. In turn, ROS could provoke signal transduction in three targets with equal probability: (i) the grafted cancer cells, (ii) the surrounding normal cells, and (iii) the surrounding extracellular matrix. This activates the integrin signaling and modulates integrin function through conformational changes [113], which is usually associated with the activation of the latent proinflammatory and profibrotic cytokines, such as TGF-β, secreted in the tumor microenvironment [114]. De Bleser et al. reported that menadione (12.5–50 μM) decreased TGF-β expression by approximately 5-fold in rat hepatic cells, and this effect correlated with a low level of glutathione, which acts as a secondary messenger in the TGF-β signal transduction pathway [115]. In our study, we observed that M/A treatment of glioblastoma-bearing mice decreased the expression of TGF-β1 by approximately 3-fold compared to the untreated mice (Figure 2J), which is direct evidence of its anti-inflammatory effect and indirect evidence of its anti-fibrotic effect. However, M/A treatment causes a significant but not very severe decrease in the oxidative capacity and inflammation of the tissues far from the primary tumor (Figure 2D,G). This suggests that there are factors other than TGF-β1 responsible for the spread of inflammation that are not affected by M/A. It should be noted that our study was conducted on T cell-immunodeficient mice, and the proinflammatory effects of TGF-β are mediated by T cell immunity [114,116]. This could also explain why the strong inhibition of TGF-β1 expression by M/A (Figure 2J) did not lead to an equally strong suppression of inflammation in the healthy tissues of our glioblastoma-bearing mice (Figure 2G). In our previous study, we demonstrated that M/A at doses achievable by oral administration significantly suppressed the expression of programmed death ligand 1 (PD-L1) in cancer cells [46]. This suggests that the anticancer effect of M/A is probably related to modulation of the immune response. PD-L1 is a transmembrane protein which is overexpressed in cancer cells and plays a major role in suppressing the adaptive immune response [117]. The PD-1/PD-L1 checkpoint pathway is one of the most promising targets of advanced cancer immunotherapy, including treatment of glioblastoma [1,13,117,118]. M/A may potentiate the effectiveness of anticancer immunotherapy and suppress the development of drug resistance, which is worth exploring in the future.

Recent studies showed that succinate is also involved in the regulation of innate immunity, the spread of inflammation, and carcinogenesis [119,120]. We observed that M/A significantly decreased the level of succinate in glioblastoma cells (Figure 3E), suggesting a decrease in their malignancy and invasiveness, as well as an anti-inflammatory effect.

Studies on wild-type animals with non-brain tumor xenografts and an intact immune system have reported that oral and parenteral M/A potentiates the efficiency of conventional chemotherapy and radiotherapy, inhibits invasion and metastasis, and increases survival [76,77,78,79,80]. The weights of the spleen and thymus were found to be higher in M/A-treated animals compared with those receiving conventional drugs alone [76,77,79], which also suggests the involvement of mechanisms related to immune stimulation. Menadione and ascorbate, administered alone, manifest anti-inflammatory effects [121,122] that also contribute to suppression of cancer progression and invasion [123]. These studies suggest that M/A may have a beneficial effect on the immune system of cancer-bearing organisms, making the cancer cells more accessible, “visible”, and perhaps more vulnerable to native immune cells. This hypothesis must be explored.

The MRI analysis of CBF and ADC provides further evidence of the versatile effects of M/A and the complexity of its selective anticancer effect (Figure 1J,K).

CBF is a marker for vascular remodeling and tissue perfusion in brain disorders, including cancer [124,125]. Poor perfusion of brain tumors, combined with severe fibrosis, maintains their hypoxic and malignant behavior without disrupting the flow of essential metabolites required for cell proliferation and invasion. The interplay between glioblastoma and the surrounding microglial cells leads to endothelial dysfunction, endothelial-to-mesenchymal transition, and neuroinflammation [126,127]. Suppression of neuroinflammation in normal brain tissue without affecting the recognition and elimination of cancer cells by the immune system is crucial to suppress glioblastoma invasion and metastasis and increase the effectiveness of conventional therapy. The access of anti-inflammatory agents to the brain and suppression of general neuroinflammation are tightly related to the cerebral blood flow. Our data demonstrate that in untreated mice, perfusion in the tumors, estimated by CBF values, was approximately four times lower than in the contralateral hemispheres (*p* < 0.01), but in M/A-treated mice, this difference was approximately two times lower (*p* < 0.05) (Figure 1J). M/A treatment of glioblastoma-bearing mice significantly increased perfusion in the tumor. Increased cerebral perfusion suggests that M/A could facilitate the penetration of conventional drugs into the tumor and potentiate their therapeutic effect. In vitro and in vivo studies have shown that M/A potentiates the efficiency of conventional chemotherapy and radiotherapy of cancer, which is often accompanied by synergistic cytotoxicity in cancer cells and suppression of tumor growth in vivo, as well as by inhibition of invasion and metastasis [46,76,77,79].

ADC is a measure of the magnitude of water diffusion in tissues [61,62]. This parameter is sensitive to the cellular density and microenvironment of the tumor [128,129]. ADC negatively correlates with compact tumor structures, characterized by a high cell density due to the proliferation of cancer cells and a “tight” microenvironment composed of fibroblasts, immune cells, and other cellular components [129]. All these cellular factors restrict the diffusion of water in the tumor. In contrast, apoptotic or necrotic areas of tumors are characterized by elevated ADC values because of the decreased cellular density [130,131]. This makes ADC a potential non-invasive biomarker for monitoring and predicting the response to therapy [129,131]. In recent years, ADC has also been used to distinguish between malignant and benign lesions [132]. Our data demonstrate that M/A treatment of glioblastoma-bearing mice significantly increased ADC values in tumors (~15%) but did not affect the values in contralateral brain tissues (Figure 1K). These findings suggest that M/A treatment decreases the cell density in the tumor and increases water diffusion, which is confirmed by histological data on M/A-treated animals published in the literature [133]. It was found that M/A (at certain doses) causes apoptosis, ferroptosis, necrosis, and a specific form of cell death called autoschizis [46,67,129,131,133]. Autoschizis is characterized by a reduction in cell size due to loss of cytoplasm by self-excision without loss of cell organelles, morphological degradation of the nucleus, and formation of apoptotic bodies [133]. During the experiments, using light microscopy, we also noticed that the size of M/A-treated glioblastoma cells markedly decreased compared to that of untreated cells, even at low/tolerable concentrations of M/A [46]. This may explain, at least partially, the decreased cellular density in M/A-treated brain tumors in vivo.

A decreased size of M/A-treated cancer cells (described in autoschizis) and a decreased cellular density in the tumor lead to an increased flow of extracellular fluids in the tumor. Such changes are not observed in non-cancerous cells and tissues. Thus, drugs and immune cells can easily reach cancer cells in M/A-treated tumors without significantly altering healthy brain tissues. Drug-induced immunomodulation and concomitant inflammation should be tightly controlled in the brain during anticancer therapy and located only in the lesion, to avoid morphological changes in healthy brain tissue and neurological disorders. Future histological/cytological experiments clarifying the effect of M/A on the level and functional state of tumor-associated macrophages and T cells can clarify the mechanism of M/A-mediated immunomodulation in glioblastoma.

## 5. Conclusions

In conclusion, our study describes a pharmacological strategy for selective targeting and treatment of glioblastoma using a redox-active combination drug, quinone/ascorbate (M/A). A single intracranial injection of M/A in mice with brain glioblastoma, and subsequent daily oral administration in the drinking water, significantly suppressed tumor growth and increased survival without adverse drug-related side effects. This was accompanied by severe induction of oxidative stress in cancer tissue, but not in healthy tissues, an increase in cerebral perfusion in the tumor, and a decrease in its cellular density.

We believe that repeated intracranial injections of M/A would stop the growth of the tumor and potentially make it operable. In a pilot study, we observed such an effect on hind paw-grafted glioblastoma mice, after three subcutaneous injections of M/A near the tumor. However, appropriate catheter system and neurosurgical procedure are needed to perform multiple intracranial injections or infusions of the drug to maintain intracranial pressure and ensure a normal brain function and a normal general condition. At present, this is difficult to achieve intracranially on small animals, but it seems possible on large animals and humans using an Ommaya reservoir.

Experimental data in vitro indicate that the mechanism of the anticancer effect of M/A is related to: (i) selective impairment of cancerous mitochondria due to a specific redox cycling between the two molecules, accompanied by a significant decrease in their over-reduced state (NADH, succinate, glutathione, etc.), mitochondrial potential, and steady-state ATP, as well as overproduction of mitochondrial superoxide; (ii) down-regulation of tNOX and TGF-β1, suggesting modulation of the immune response and a potential role in suppressing drug resistance.

In recent decades, we have learned that targeting a single enzyme or pathway rarely leads to successful cancer treatment. Specific redox targeting of cancer cells based on their altered mitochondrial bioenergetics and metabolism is emerging as a promising and safe new strategy for successful cancer therapy due to its universality in transformed cells (so-called “mitocan therapy”) [134]. Altered mitochondrial function, characterized by their over-reduced (overcharged) Q-pools, and overloading with metabolites (such as succinate and NADH), is a prominent feature of all cancers and potentially a root cause of their proliferation and escape from the immune system. It appears that M/A can “recognize” altered mitochondria and selectively impair their “pro-oncogenic” functionality. This may alter T cell differentiation and/or “mark” cancer cells to make them more “visible” and “accessible” to the immune system and conventional therapies. Nature has selected these redox-active substances as essential during evolution (menadione (pro-vitamin K3) and ascorbate (vitamin C)). Perhaps their main non-vitamin function is to detect defective cells, to “mark” them by altering their redox homeostasis, and to facilitate mechanisms for their removal. In this context, safe redox-active compounds such as M/A and other quinone/ascorbate combinations, acting by the described redox-targeting mechanism, could significantly contribute to the success of therapy in glioblastoma, as well as other cancers. Defective mitochondria appear to be an “Achilles’ heel” for the M/A combination and a major molecular target for its anticancer effect.

## Data Availability

Data are contained within the article.

## Supplementary Materials

The following supporting information can be downloaded at: https://www.mdpi.com/article/10.3390/cancers14030485/s1, Figure S1: Schematic representation of redox cycling of menadione with production of superoxide and hydrogen peroxide; Figure S2: Experimental design on glioblastoma mice; Figure S3: Microscopic detection of thrombosis before and after intravenous administration of M/in healthy mice; Figure S4: Concentration-dependent and time-dependent effects of M/A on proliferation and viability of temozolomide-treated glioblastoma cells; Table S1: Redox therapies against glioblastoma.
