# Supplementary material for: Targeting Glioblastoma via Selective Alteration of Mitochondrial Redox State
Source: Cancers (Basel). 2022 Jan 19;14(3):485. doi: 10.3390/cancers14030485 (PMC8833725; doi:10.3390/cancers14030485)
Supplement: Supplementary file 1 [file cancers-14-00485-s001.zip › cancers-1519153-SI.pdf]

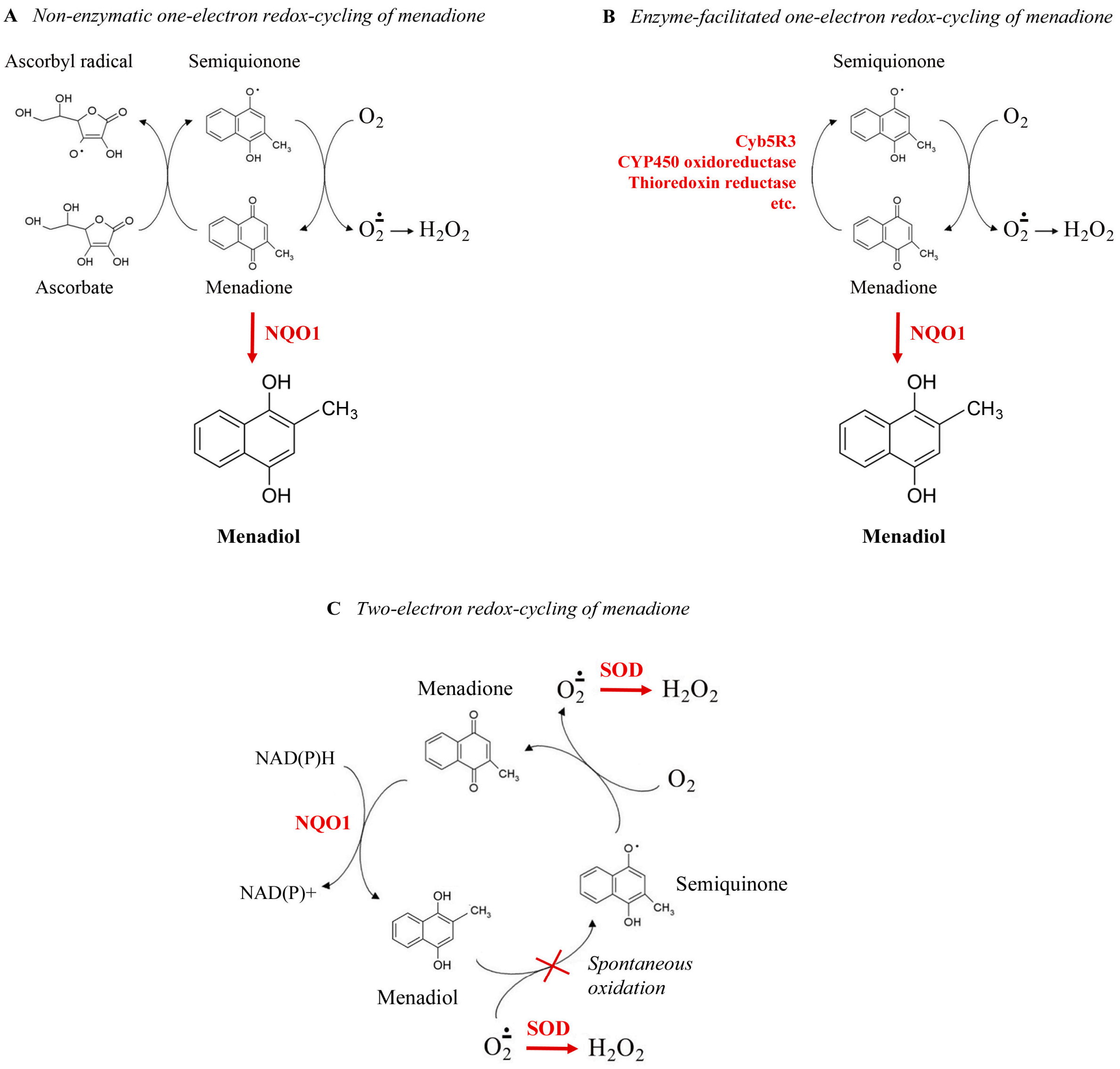

Figure S1. Schematic representation of redox-cycling of menadione with production of superoxide and hydrogen peroxide [1–7]: (A) non-enzymatic ascorbate-driven one-electron redox-cycling; (B) enzyme-facilitated one-electron redox-cycling; (C) two-electron redox-cycling by NQO1 and subsequent autooxidation.CYP450 – cytochrome P-450, Cyb5R3 – NADH-cytochrome b5 oxidoreductase 3; NQO1 – NAD(P)H-dehydrogenase quinone 1; SOD – superoxide dismutase.NQO1 maintains menadione in its reduced form (menadiol) and thus depletes the menadione required for one-electron redox-cycling mechanisms, while SOD converts superoxide into hydrogen peroxide, thus inhibiting spontaneous oxidation of menadiol to semiquinone.

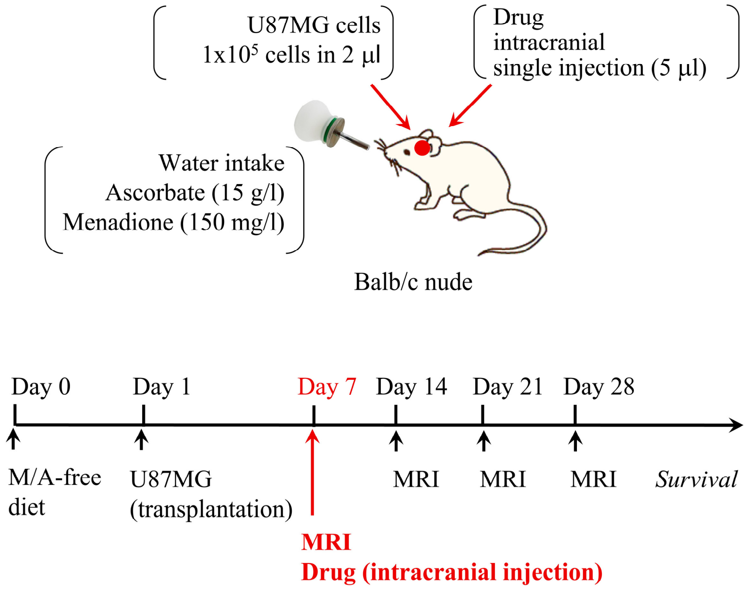

Figure S2. Experimental design on U87MG glioblastoma mice: Treatment of mice with menadione/ascorbate (M/A) or saline solution (Control).

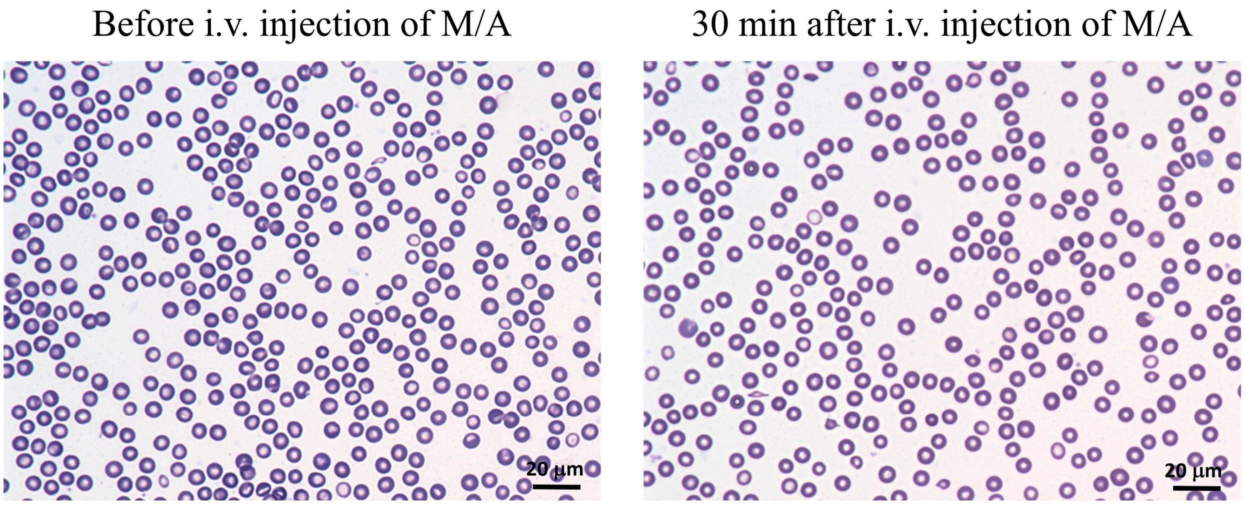

Figure S3. Microscopic detection of thrombosis before and 30 min after intravenous administration of M/A (140 µg/14 mg per kg body weight) in healthy mice (a Romanowsky staining). Aggregates were not detected.

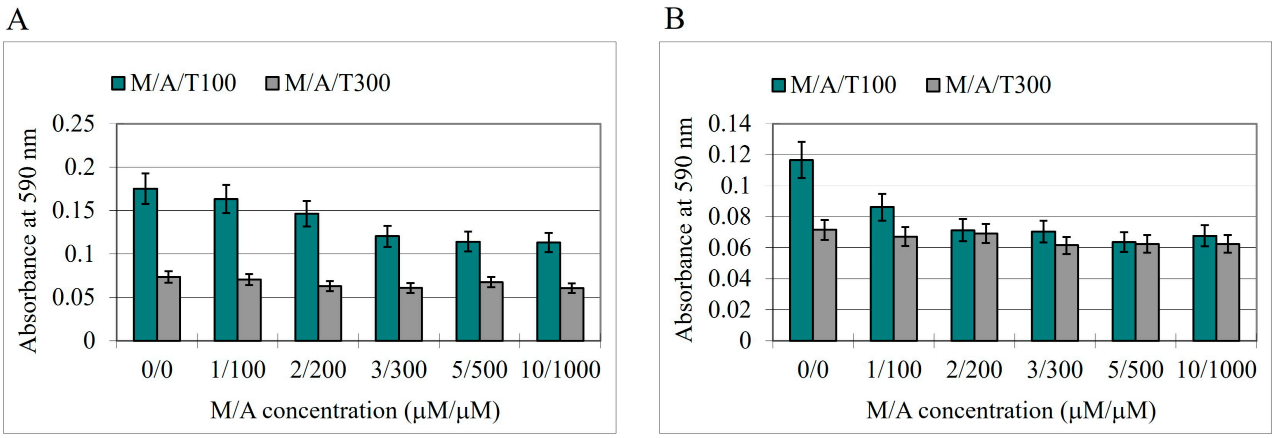

Figure S4. Concentration-dependent and time-dependent effects of M/A on proliferation and viability of temozolomide-treated glioblastoma cells (U87MG): (A) 24-hours of incubation; (B) 48-hours of incubation. Green columns (T100) – 100 µM temozolomide; Gray columns (T300) – 300 µM temozolomide. MTT test was used for analysis. In (A), the absorbance of untreated control was 0.38; In (B), the absorbance of untreated control was 0.61. Initial number of cells in all samples was $0.6x10^5$ cells per well. Data are means±SD from three independent experiments with two parallel measurements for each experiment.

Table S1. Redox therapies against glioblastoma [adapted by Salazar-Ramiro et al. [8] and supplemented].

Anticancer Compound *	Redox Effect	References
Chloroquine	↑ ROS/RNS<br>↑ Mitochondrial superoxide<br>↓ Mitochondrial membrane potential<br>↓ Glutathione	[9–11]
t-BOOH (tertiary-buthylhydroperoxide)	↑ ROS<br>↓ Glutathione	[12]
OGD<br>(oxygen-glucose deprivation)	↑ ROS<br>↓ Mitochondrial membrane potential	[13,14]
Quercetin	↑ ROS<br>↓ Mitochondrial membrane potential	[15,16]
Rutin	↑ ROS<br>↓ Mitochondrial membrane potential	[16]
EGGG<br>(epigallocatechin-3-gallate	↑ ROS<br>↓ Mitochondrial membrane potential	[17,18]
PITC<br>(phenylethyl isothiocyanate)	↑ ROS<br>Mitochondrial dysfunction	[19]
Gambogic acid	↑ ROS<br>Mitochondrial dysfunction	[20]
Artocarpesin, Cycloartocarpesin,<br>Isobavachalcone	↑ ROS<br>↓ Mitochondrial membrane potential	[21]
Pt-1-DMCa (platimun analog)	↑ ROS	[22]
Arecaidine propargyl ester	↑ ROS	[23]
Cannabidiol	↑ ROS<br>↓ Mitochondrial membrane potential	[24]
Oligomeric procyanidins	↑ ROS<br>↓ Mitochondrial membrane potential	[25]
Alantolactine	↑ ROS<br>↓ Mitochondrial membrane potential<br>↓ Glutathione	[26]

Bromopyruvate	↑ ROS<br>↓ Mitochondrial membrane potential	[27]
Manganese porphyrin	↑ ROS/RNS	[28]
Apigenin, Epigallocatechin, Genistein	↑ ROS<br>↓ Mitochondrial membrane potential	[29]
Kaempferol	↑ ROS<br>↓ Mitochondrial membrane potential<br>↓ Thioredoxin	[29]
PENAO<br>[4-N-(S-penicillaminylacetyl) amino) phenylarsonous acid] – alone and combination	↑ ROS<br>↑ Mitochondrial ROS<br>↓ Mitochondrial membrane potential<br>↓ Oxygen consumption rate	[30]
DCA<br>(sodium dichloroacetate)	↑ ROS<br>↑ Mitochondrial ROS<br>↓ Mitochondrial membrane potential	[31]
Ascorbic acid (5-100 mM)	↑ ROS	[32]
Xanthohumol	↑ ROS<br>↓ Mitochondrial membrane potential	[33]
Berberine	↑ ROS<br>↓ Mitochondrial membrane potential	[34]
Buthionine sulfoximine	↑ ROS<br>↓ Glutathione	[35,36]
Menadione/Ascorbate (M/A)	↑ ROS<br>↑ Mitochondrial ROS<br>↓ Mitochondrial membrane potential<br>↓ NADH, NAD^+<br>↓ Succinate<br>↓ Glutathione<br>↓ Thioredoxin (reduced)<br>↓ Ribonucleotide reductase	[37–41]

* All compounds are found to induce apoptosis and cytotoxicity towards glioblastoma cells

References

1. Nikiforova, A.B.; Saris, N.-E.L.; Kruglov, A.G. External Mitochondrial NADH-Dependent Reductase of Redox Cyclers: VDAC1 or Cyb5R3? *Free Radic. Biol. Med.* **2014**, *74*, 74–84. https://doi.org/10.1016/j.freeradbiomed.2014.06.005.
2. Beck, R.; Pedrosa, R.C.; Dejenas, N.; Glorieux, C.; Gallez, P.L.B.; Taper, H.; Eeckhoudt, S.; Knoops, L.; Calderon, P.B.; Verrax, J. Ascorbate/Menadione-Induced Oxidative stress Kills Cancer Cells That Express Normal or Mutated Forms of the Oncogenic Protein Bcr-Abl. An In Vitro and In Vivo Mechanistic Study. *Invest. New Drugs* **2011**, *29*, 891–900. https://doi.org/10.1007/s10637-010-9441-3.
3. Glorieux, C.; Calderon, P.B. Cancer Cell Sensitivity to Redox-Cycling Quinones Is Influenced by NAD(P)H: Quinone Oxidoreductase 1 Polymorphism. *Antioxidants* **2019**, *8*, 369. https://doi.org/10.3390/antiox8090369
4. Jabarak, R.; Jabarak, J. Effect of Ascorbate on the DT-diaphorase-mediated Redox Cycling of 2-methyl-1,4-naphthoquinone. *Arch. Biochem. Biophys*. **1995**, *318*, 418–423, https://doi.org/10.1006/abbi.1995.1249
5. Keller, M.A.; Piedrafita, G.; Ralser, M. The Widespread Role of Non-enzymatic Reactions in Cellular Metabolism. *Curr. Opin. Biotechnol*. **2015**, *34*, 153–161, https://doi.org/10.1016/j.copbio.2014.12.020
6. Li, J.; Zuo, X.; Cheng, P.; Ren, X.; Sun, S.; Xu, J.; Holmgren, A.; Lu, J. The Production of Reactive Oxygen Species Enhanced with the Reduction of Menadione by Active Thioredoxin Reductase. *Metallomics* **2019**, *11*, 1490–1497, https://doi.org/10.1039/C9MT00133f.
7. Gray, J.P.; Karandrea, S.; Burgos, D.Z.; Jaiswal, A.A.; Heart, E.A. NAD(P)H-dependent Quinone Oxidoreductase 1 (NQO1) and Cytochrome P450 Oxydoreductase (CYP450OR) Differentially Regulate Menadione-mediated Alterations in Redox Status, Survival and Metabolism in Pancreatic Beta-cells. *Toxicol. Lett*. **2016**, *262*, 1–11, https://doi.org/10.1016/j.toxlet.2016.08.021

8. Salazar-Ramiro, A.; Ramirez-Ortega, D.; de la Cruz, V.P.; Hernandez-Pedro, N.Y.; Gonzalez-Esquivel, D.F.; Sotelo, J.; Pineda, B. Role of Redox Status in Development of Glioblastoma. *Front. Immunol.* **2016**, *7*, 156. https://doi.org/10.3389/fimmu.2016.00156.
9. Chen T.H.; Chang P.C.; Chang M.C.; Lin Y.F.; Lee H.M. Chloroquine Induces the Expression of Inducible Nitric Oxide Synthase in C6 Glioma Cells. *Pharmacol. Res.* **2005**, *51*, 329–336. https://doi.org/10.1016/j.phrs.2004.10.004.
10. Vessoni A.T.; Quinet A.; Andrade-Lima L.C.; Martins D.J.; Garcia C.C.; Rocha C.C.R.; Vieira D.B.; Menck C.F.M. Chloroquine-induced Glioma Cells Death Is Associated with Mitochondrial Membrane Potential Loss, but Not Oxidative Stress. *Free Radic. Biol. Med.* **2016**, *90*, 91–100. https://doi.org/10.1016/j.freeradbiomed.2015.11.008.
11. Park B.C.; Park S.H.; Paek S.H.; Park S.Y.; Kwak M.K.; Choi H.G.; Yong C.S.; Yoo B.K.; KimJ.-A. Chloroquine-induced Nitric Oxide Increase and Cell Death Is Dependent on Cellular GSH Depletion in A172 Human Glioblastoma Cells. *Toxicol Lett.* **2008**, *178*, 52–60. https://doi.org/10.1016/j.toxlet.2008.02.003.
12. Gitika B.; Sai Ram M.; Sharma S.K.; Ilavazhagan G.; Banerjee P.K. Quercetin Protects C6 Glial Cells from Oxidative Stress Induced by Tertiary-butylhydroperoxide. *Free Radic. Res.* **2006**, *40*, 95–102. https://doi.org/10.1080/10715760500335447.
13. Panickar K.S.; Anderson R.A. Mechanisms Underlying the Protective Effects of Myricetin and Quercetin Following Oxygen-glucose Deprivation-induced Cell Swelling and the Reduction in Glutamate Uptake in Glial Cells. *Neuroscience*. **2011**, *183*, 1–14. https://doi.org/10.1016/j.neuroscience.2011.03.064.
14. Vidak M.; Rozman D.; Komel R. Effects of Flavonoids from Food and Dietary Supplements on Glial and Glioblastoma Multiforme Cells. *Molecules* **2015**, *20*, 19406–19432. https://doi.org/10.3390/molecules201019406.
15. Jakubowicz-Gil J.; Langner E.; Badziul D.; Wertel I.; Rzeski W. Apoptosis Induction in Human Glioblastoma Multiforme T98G Cells upon Temozolomide and Quercetin Treatment. *Tumour Biol.* **2013**, *34*, 2367–2378. https://doi.org/10.1007/ s13277-013-0785-0.
16. Imani A.; Malek N.; Bohlouli S.; Kouhsoltani M.; Sharifi S.; Dizaj S.M. Molecular Mechanisms of Anticancer Effect of Rutin. *Phytother. Res.* **2020**, E-pub: December 9. https://doi.org/10.1002/ptr.6977.
17. Agarwal A.; Sharma V.; Tewari R.; Koul N.; Joseph C.; Sen E.; Epigallocatechin-3-gallate Exhibits Anti-tumor Effect by Perturbing Redox Homeostasis, Modulating the Release of Pro-inflammatory Mediators and Decreasing the Invasiveness of Glioblastoma Cells. *Mol. Med. Rep.* **2008**, *1*, 511–515. https://doi.org/10.3892/mmr.1.4.511.
18. Das A.; Banik N.L.; Ray S.K. Flavonoids Activated Caspases for Apoptosis in Human Glioblastoma T98G and U87MG Cells but Not in Human Normal Astrocytes. *Cancer* **2010**, *116*, 164–176. https://doi.org/10.1002/cncr.24699.
19. Chou Y.C.; Chang M.Y.; Wang M.J.; Liu H.C.; Chang S.J; Harnod T.; et al. Phenethyl Isothiocyanate Alters the Gene Expression and the Levels of Protein Associated with Cell Cycle Regulation in Human Glioblastoma GBM 8401 Cells. *Environ. Toxicol.* **2017**, *32*, 176–187. https://doi.org/10.1002/tox.22224.
20. Xu J.; Zhou M.; Ouyang J.; Wang J.; Zhang Q.; Xu Y.; Zhang Q.; Xu X.; Zeng H. Gambogic Acid Induces Mitochondria-dependent Apoptosis by Modulation of Bcl-2 and Bax in Mantle Cell Lymphoma JeKo-1 Cells. *Chin. J. Cancer Res.* **2013**, *25*, 183–191. https://doi.org/10.3978/j.issn.1000-9604.2013.02.06.
21. Kuete V.; Mbaveng A.T.; Zeino M.; Fozing C.D.; Ngameni B.; Kapche G.D.W.; Ngadjui B.T. Cytotoxicity of Three Naturally Occurring Flavonoid Derived Compounds (Artocarpesin, Cycloartocarpesin and Isobavachalcone) towards Multi-factorial Drug-resistant Cancer Cells. *Phytomedicine* **2015**, *22*, 1096–1102. https://doi.org/10.1016/j.phymed.2015.07.006.
22. Aroui S.; Dardevet L.; Ajmia W.B.; de Boisvilliers M.; Perrin F.; Laajimi A.; Boumendjel A.; Kenani A.; Muller J.-M.; De Waard M. A Novel Platinum-maurocalcine Conjugate Induces Apoptosis of Human Glioblastoma Cells by Acting through the ROS-ERK/AKT-p53 Pathway. *Mol. Pharm.* **2015**, *12*, 4336–4348. 10.1021/acs.molpharmaceut.5b00531.
23. Di Bari M.; Tombolillo V.; Conte C.; Castigli E.; Sciaccaluga M.; Iorio E.; Carpinelli G.; Ricordy R.; Fiore M.; Degrassi F.; et al. Cytotoxic and Genotoxic Effects Mediated by M2 Muscarinic Receptor Activation in Human Glioblastoma Cells. *Neurochem. Int.* **2015**, *90*, 261–270. https://doi.org/10.1016/j.neuint.2015.09.008
24. Chan J.Z.; Duncan R.E. Regulatory Effects of Cannabidiol on Mitochondrial Functions: A Review. *Cells* **2021**, *10*, 1251. https://doi.org/10.3390/cells10051251.
25. Zhang F.J.; Yang J.Y.; Mou Y.H.; Sun B.S.; Wang J.M.; Wu C.F. Oligomer Procyanidins from Grape Seeds Induce a Paraptosis-like Programmed Cell Death in Human Glioblastoma U-87 Cells. *Pharm. Biol.* **2010**, *48*, 883–890. https://doi.org/10.3109/13880200903311102
26. Khan M.; Yi F.; Rasul A.; Li T.; Wang N.; Gao H.; Gao R.; Ma T. Alantolactone Induces Apoptosis in Glioblastoma Cells via GSH Depletion, ROS Generation, and Mitochondrial Dysfunction. *IUBMB Life* **2012**, *64*, 783–794. https://doi.org/10.1002/iub.1068.

27. Macchioni L.; Davidescu M.; Sciaccaluga M.; Marchetti C.; Migliorati G.; Coaccioli S.; Castigli E. Mitochondrial Dysfunction and Effect of Antiglycolytic Bromopyruvic Acid in GL15 Glioblastoma Cells. *J. Bioenerg. Biomembr*. **2011**, *43*, 507–518. https://doi.org/10.1007/s10863-011-9375-2.
28. Keir S.T.; Dewhirst M.W.; Kirkpatrick J.P.; Bigner D.D.; Batinic-Haberle I. Cellular Redox Modulator, Ortho Mn(III) Meso-tetrakis(N-n-hexylpyridinium-2-yl)Porphyrin, MnTnHex-2-PyP(5+) in the Treatment of Brain Tumors. *Anticancer Agents Med. Chem*. **2011**, *11*, 202–212. https://doi.org/10.2174/187152011795255957.
29. Sharma V.; Joseph C.; Ghosh S.; Agarwal A.; Mishra M.K.; Sen E. Kaempferol Induces Apoptosis in Glioblastoma Cells through Oxidative Stress. *Mol. Cancer Ther*. **2007**, *6*, 2544–2553. https://doi.org/10.1158/1535-7163.MCT-06-0788.
30. Shen H.; Decollogne S.; Dilda P.J.; Hau E.; Chung S.A.; Luk P.P.; Hogg P.J.; McDonald K.L. Dual-targeting of Aberrant Glucose Metabolism in Glioblastoma. *J. Exp. Clin. Cancer Res*. **2015**, *34*, 14. https://doi.org/10.1186/s13046-015-0130-0.
31. Michelakis E.D.; Sutendra G.; Dromparis P.; Webster L.; Haromy A.; Niven E.; Maguire C.; Gammer T.-L.; Mackey J.R.; Fulton D.; et al. Metabolic Modulation of Glioblastoma with Dichloroacetate. *Sci. Transl. Med*. **2010**, *2*, 31ra34. https://doi.org/10.1126/scitranslmed.3000677.
32. Klingelhoeffer C.; Kammerer U.; Koospal M.; Muhling B.; Schneider M.; Kapp M.; Kübler A.; Germer C.-T.; Otto C. Natural Resistance to Ascorbic Acid Induced Oxidative Stress Is Mainly Mediated by Catalase Activity in Human Cancer Cells and Catalase-silencing Sensitizes to Oxidative Stress. *BMC Complement. Altern. Med*. **2012**, *12*, 61. https://doi.org/10.1186/1472-6882-12-61.
33. Zhang B.; Chu W.; Wei P.; Liu Y.; Wei T. Xanthohumol Induces Generation of ROS and Triggers Apoptosis through Inhibition of Mitochondrial Electron-transport Chain Complex I. *Free Radic. Bio. Med*. **2015**, *89*, 486-497. https://doi.org/10.1016/j.freeradbiomed.2015.09.021
34. Eom K.S.; Kim H.J.; So H.S.; Park R., Kim T.Y. Berberine-induced Apoptosis in Human Glioblastoma T98G Cells Is Mediated by Endoplasmic Reticulum Stress Accompanying Reactive Oxygen Species and Mitochondrial Dysfunction. *Biol. Pharm. Bull*. **2010**, *33*, 1644-1649. https://doi.org/10.1248/bpb.33.1644
35. Iida M.; Doi H.; Asamoto S.; Sugiyama H.; Sakagami H.; Kuribayashi N.; Takeda M.; Okamura Y.; Matsumoto K. Effect of Glutathione-modulating Compounds on Platinum Compounds-induced Cytotoxicity in Human Glioma Cell Lines. *Anticancer Res*. **1999**, *19*, 5383–5384.
36. Iida M.; Sunaga S.; Hirota N.; Kuribayashi N.; Sakagami H.; Takeda M.; Matsumoto K. Effect of Glutathione-modulating Compounds on Hydrogen-peroxide-induced Cytotoxicity in Human Glioblastoma and Glioma Cell Lines. *J. Cancer Res. Clin. Oncol*. **1997**, *123*, 619–622. https://doi.org/10.1007/s004320050115.
37. Berraondo, P.; Sanmamed, M.F.; Ochoa, M.C.; Etxeberria, I.; Aznar, M.A.; Perez-Gracia, J.L.; Rodriguez-Ruiz, M.E.; Ponz-Sarvise, M.; Castanon, E.; Melero, I. Cytokines in Clinical Cancer Immunotherapy. *Brit. J. Cancer* **2019**, *120*, 6–15. https://doi.org/10.1038/s41416-018-0328-y.
38. The Human Protein Atlas: ENOX2 Protein Expression Summary. Available online: http://www.proteinatlas.org/ ENSG00000165675-ENOX2 (accessed on 2 November 2021).
39. Ryszawy, D.; Pudelek, M.; Catapano, J.; Ciarach, M.; Setkowicz, Z.; Konduracka, E.; Madeja, Z.; Czyz, J. High Doses of Sodium Ascorbate Interfere with the Expansion of Glioblastoma Multiform Cells In Vitro and In Vivo. *Life Sci.* **2019**, *232*, 116657. https://doi.org/10.1016/j.lfs.2019.116657.
40. Padayatty, S.; Sun, A.Y.; Chen, Q.; Epsey, M.G.; Drisko, J.; Levine, M. Vitamin C: Intravenous Use by Complementary and Alternative Medicine Practitioners and Adverse Effects. *PLoS ONE* **2010**, *5*, e11414. https://doi.org/10.1371/journal.pone.0011414.
41. Carr, A.C.; Cook, J. Intravenous Vitamin C for Cancer Therapy—Identifying the Current Gaps in Our Knowledge. *Front. Physiol.* **2018**, *9*, 1182. https://doi.org/10.3389/fphys.2018.01182.
